# From Coprecipitation to Nanostructure: Formation, Morphology, and Reactive-Site Accessibility in Fe-Containing Binary Layered Double Hydroxides

**DOI:** 10.3390/nano16191210

**Published:** 2026-09-24

**Authors:** Alua Alikeyeva, Nurbolat Kudaibergenov, Kairzhan Shalmagambetov, Abzal Azimbay

**Affiliations:** 1 Center of Physical Chemical Methods of Research and Analysis, Al-Farabi Kazakh National University, Almaty 050040, Kazakhstan; 2 Department of Chemistry, Kazakh National Women’s Teacher Training University, Almaty 050000, Kazakhstan

**Keywords:** layered double hydroxide, Fe-LDH, coprecipitation, phase formation, crystal chemistry, nanostructure, reactive-site accessibility, green rust

## Abstract

Binary Fe-containing LDHs and structurally related layered hydroxides are being widely investigated as adsorbents, catalysts, and redox-active nanomaterials. Yet, links among coprecipitation, phase formation, nanostructure, and reactive-site accessibility remain inconsistently interpreted. This critical review evaluates studies published mainly from 2015 to July 2026 on CoFe, NiFe, MgFe, ZnFe, MnFe, CuFe, and CaFe layered hydroxides and Fe(II)/Fe(III) green rust. Coprecipitation is not a single standardized route: local supersaturation, reagent delivery, mixing, complexation, atmosphere, interlayer chemistry, and aging can alter metal incorporation and phase development, while Fe-rich transient precursors may participate under system-specific conditions. The commonly cited Fe(III) fraction of x = 0.20–0.33 is an empirical guideline rather than a universal stability window, and conventional M(II)-Fe(III) LDHs, CaFe hydrocalumite-/AFm-related phases, and green rust require distinct crystal-chemical interpretations. Nominal composition and LDH-like diffraction cannot establish homogeneous cation incorporation or phase purity. In contrast, diffraction broadening, BET area, nanosheet dimensions, and XPS fitting do not provide stand-alone evidence of defects or accessible reactive sites. Progress toward predictive synthesis requires standardized reporting, system-specific synthesis–composition–phase maps, time-resolved studies, quantitative structure–accessibility relationships, and recognition of as-synthesized, working, recovered, and regenerated materials as potentially distinct structural states, supported by uncertainty analysis, negative outcomes, and validation across laboratories.

## 1. Introduction

Layered double hydroxides (LDHs) consist of positively charged metal hydroxide layers separated by charge-compensating interlayer anions and water molecules. Their general composition is commonly expressed as:

$${\left[M_{1-x\;}^{II}M_{x\;}^{III}{\;(OH)}_{2}\right]}^{x+}\cdot {\left(A^{n-}\right)}_{x/n}\cdot mH_{2}O$$

where *M^II^* and *M^III^* are divalent and trivalent metal cations, respectively, *A^n−^* represents an interlayer anion, and *mH*_2_*O* represents interlayer water. The partial substitution of divalent cations by trivalent cations generates a positive layer charge, which is balanced by anions located in the interlayer region [1,2].

The adjustable composition of the hydroxide layers and interlayer region makes LDHs useful as adsorbents, ion exchangers, catalysts, photocatalysts, electrocatalysts, and mixed-metal oxide precursors. Their behavior depends on the combined effects of metal composition, interlayer chemistry, surface hydroxylation, nanostructure, and structural evolution under given operating conditions [3,4,5].

LDHs should not, however, be treated as a uniform material class. Products assigned to the same nominal system may differ in actual metal incorporation, phase composition, crystallinity, aggregation, and surface chemistry because of variations in synthesis and post-synthetic treatment [6,7,8]. This variability is especially important for binary Fe-containing layered hydroxides, in which Fe hydrolysis, oxidation state, local coordination, and interaction with the second metal influence phase formation and stability [9,10,11,12,13,14,15].

Binary Fe-containing systems therefore provide a useful basis for examining how precipitation history controls the composition, phase identity, nanostructure, and accessibility of reactive sites. Their comparison nevertheless requires phase-specific interpretation because conventional M(II)-Fe(III) LDHs, CaFe layered hydroxides, and Fe(II)/Fe(III) green rust differ in crystal chemistry and formation behavior.

In this review, the term “reactive-site environment” refers to a structurally or chemically plausible local environment that may participate in adsorption, redox, catalytic, or electrochemical processes, including accessible metal-centered hydroxyl sites, undercoordinated edge or termination sites, defect-associated environments, interlayer-accessible regions, and redox-active Fe/M centers. This distinction separates the structural presence of such environments from their physical and chemical accessibility and their experimentally demonstrated activity under specific operating conditions [11]. The present review primarily addresses the first two aspects. Accordingly, morphology, BET surface area, diffraction peak broadening, or fitted XPS components are treated as structural descriptors rather than direct evidence of catalytic or adsorption active sites. Robust assignment of an operationally active site therefore requires application-specific measurements, supported where possible by site-sensitive characterization and in situ/operando or other mechanistically discriminating evidence.

### 1.1. Binary Fe-Containing Layered Double Hydroxides as Nanostructured Functional Materials

In this review, the term “LDH nanomaterials” refers to LDH materials composed of nanoscale platelets, nanoplatelets, or nanosheets with at least one external dimension within the 1–100 nm range [16]. These primary units may assemble into larger aggregates or hierarchical architectures, so a microscale secondary particle can still be nanostructured if it is built from nanoscale LDH units [17,18].

The properties of binary Fe-containing layered hydroxides are controlled at several interconnected levels, including metal composition and distribution, interlayer anion chemistry, platelet dimensions, aggregation, surface hydroxylation, local disorder, redox heterogeneity, and structural transformation. These features determine the accessibility and chemical state of layer edges, interlayer entrances, surface sites, and reconstructed domains [19,20,21,22,23,24,25,26,27,28,29]. They are examined in detail through crystal chemistry and Fe speciation in Section 2, precipitation and post-synthetic control in Section 3, and nanostructure, accessibility, defects, and material-state evolution in Section 4. Section 5 integrates the resulting knowledge gaps and future research priorities.

### 1.2. Why Coprecipitation Remains the Principal Synthesis Route

Several methods have been developed for LDH preparation, including coprecipitation, homogeneous precipitation, hydrothermal and solvothermal syntheses, electrodeposition, mechanochemical synthesis, sol–gel processing, and reconstruction of calcined mixed oxides [6,18]. These routes can improve crystallinity, film formation, morphology control, or solvent economy in specific cases, but they differ strongly in equipment requirements, reaction environment, scalability, and product form [18,30,31,32,33,34].

For this review, coprecipitation was selected as the common synthesis framework because it is widely used, experimentally simple, compositionally flexible, and applicable to most binary Fe-containing LDH powders [35,36]. It also allows the comparison of major chemical variables such as pH, metal ratio, reagent addition, aging, atmosphere, and interlayer anion identity across systems [37,38].

These variables influence Fe hydrolysis, supersaturation, metal incorporation, intermediate-phase formation, and final phase identity for Fe-containing systems [38,39,40]. Coprecipitation should therefore not be regarded as a single standardized method or assumed to be reproducible from nominal recipes alone [41]. The effects of different precipitation histories on structure and morphology are examined systematically in Section 3.

### 1.3. Scope, Selection Criteria, and Objectives of the Review

This review focuses primarily on binary M-Fe layered double hydroxides prepared by coprecipitation, together with structurally related CaFe layered hydroxides and Fe(II)/Fe(III) green rust included for phase-specific comparison. The systems considered are Co-Fe, Ni-Fe, Mg-Fe, Zn-Fe, Mn-Fe, Cu-Fe, Ca-Fe, and Fe(II)/Fe(III) layered hydroxides. Environmental and electrochemical applications provide the broader motivation for studying these materials, but the present review concentrates on synthesis–structure relationships rather than application-specific performance.

In this review, “binary M-Fe LDHs” refers to hydroxide layers containing Fe and one additional principal metal component. Fe(II)/Fe(III) green rust is not a binary M-Fe system in this compositional sense but is included as a structurally related mixed-valent Fe layered hydroxide because its charged hydroxide layers and interlayer anion chemistry provide a mechanistically informative comparison with conventional M(II)-Fe(III) LDHs [2]. CaFe layered hydroxides are likewise considered separately because their crystal chemistry differs from that of conventional brucite-like M(II)-Fe(III) LDHs [42].

Accordingly, the reviewed materials are evaluated within three structural families: conventional M(II)-Fe(III) LDHs, CaFe layered hydroxides, and Fe(II)/Fe(III) green rust. This classification prevents their compositional limits, phase identity, redox chemistry, and transformation behavior from being interpreted through a single generalized LDH model.

The literature coverage was evaluated through a structured literature-search strategy using Scopus and Web of Science Core Collection as the principal bibliographic databases, supplemented by Google Scholar and, where relevant, PubMed. The principal search covered publications from 2015 to 2026 and combined terms related to layered double hydroxides (“layered double hydroxide”, “LDH”, “hydrotalcite”, “green rust”, and “hydrocalumite”) with Fe-containing compositions and terms describing synthesis, coprecipitation, formation mechanisms, structural evolution, morphology, anion exchange, aging, and characterization. The final search update was conducted in July 2026, with database queries applied primarily to titles, abstracts, and keywords. Earlier publications were retained when they represented foundational contributions to LDH nomenclature, structural chemistry, formation mechanisms, phase stability, or characterization methodology. Reference lists of relevant reviews and primary studies were additionally screened to identify pertinent earlier or otherwise missed publications.

Studies were included when they provided experimentally relevant information linking synthesis or post-synthetic conditions to composition, phase identity, formation pathway, morphology, aggregation, porosity, defect chemistry, redox state, or reactive-site accessibility. Particular weight was given to studies reporting measured solid composition, complementary phase-sensitive characterization, controlled comparisons of synthesis parameters, or time-resolved, in situ, or operando evidence. Studies reporting only functional performance without sufficient synthesis or structural characterization were not used as primary evidence for synthesis–structure relationships.

The principal analysis was restricted to the Fe-containing layered hydroxide systems defined above and prepared by coprecipitation or closely related precipitation routes. Multimetal LDHs, supported composites, calcined products, reconstructed materials, and materials prepared by substantially different synthesis routes were considered only when they provided direct mechanistic or comparative evidence relevant to a binary Fe-containing precursor [43]. Studies with ambiguous phase assignment or insufficient experimental detail were used, where necessary, only as supporting observations and were not considered sufficient on their own to establish general compositional windows, phase-purity criteria, or formation mechanisms [44]. Forward citation searching of key mechanistic studies was also used to identify newer confirmatory or contradictory evidence.

The objectives of this review are to compare how coprecipitation variables control hydrolysis, nucleation, metal incorporation, aging, and phase formation across the Fe-containing systems. Particular attention is given to system-specific compositional and phase-purity criteria and to the evidence supporting direct co-nucleation, precursor-mediated growth, dissolution–reprecipitation, and redox-controlled transformation. The review further examines how synthesis affects nanostructure and reactive-site accessibility, distinguishes initial materials from their active or transformed states, and identifies priorities for standardized reporting, reproducibility, time-resolved characterization, and quantitative synthesis–structure relationships.

Application-dependent removal mechanisms, realistic water matrices, regeneration, toxicity, material recovery, and practical implementation are outside the principal scope of this synthesis-focused review and will be addressed separately in a companion Part II.

Several reviews have addressed the synthesis, structure, and environmental applications of LDH-based materials from different perspectives. Broad environmental reviews have summarized LDHs, LDH-derived oxides, and their composites according to synthesis method, pollutant class, or treatment process [45]. More specialized studies have examined the formation pathways of M(II)/M(III) LDHs [46], the characterization and variable-pH coprecipitation mechanisms of Al(III)- and Fe(III)-based LDHs [38], and the synthesis, redox properties, and water-decontamination performance of Fe(II)-Fe(III) green rust [26]. A recent review has also emphasized the general relationships among LDH synthesis, structural properties, and environmental performance [47].

These contributions establish important general principles but generally address either the wider LDH family, selected formation mechanisms, or individual classes of Fe-containing materials. A systematic composition-resolved comparison of the major binary Fe-containing layered hydroxide systems within a common synthesis–formation–nanostructure framework remains limited.

The novelty of the present review lies in comparing eight Fe-containing layered hydroxide systems using common criteria while retaining their distinct structural chemistry. Rather than treating all materials through a single generic LDH model, the analysis distinguishes conventional brucite-like M(II)-Fe(III) LDHs, hydrocalumite- or AFm-related CaFe layered hydroxides, and mixed-valent Fe(II)/Fe(III) green rust. Particular attention is given to the strength of evidence supporting phase identity and formation pathways, including whether the final layered phase forms directly or through Fe-rich intermediates, dissolution–reprecipitation, aging-induced reorganization, or redox-mediated transformation.

The review further separates nominal precursor composition from measured solid composition, dry-state physical area from chemically accessible reactive sites, and the as-synthesized material from its active, reconstructed, and recovered states. By integrating composition, precipitation history, formation pathways, nanostructure, phase-sensitive characterization, and material-state evolution, the review aims to establish a more rigorous basis for interpreting synthesis–structure relationships in binary Fe-containing layered hydroxides (Figure 1). Figure 1 summarizes this framework as a six-step sequence, progressing from coprecipitation history and early hydrolysis/intermediate formation to phase identity and composition, nanostructure and aggregation, reactive-site environment and accessibility, and ultimately the operational and recovered state of the material. Importantly, the framework separates structural descriptors and candidate reactive-site environments from experimentally demonstrated activity, while also recognizing that the material present during or after operation may differ from the as-synthesized layered phase.

## 2. Structural Chemistry and Formation of Binary M-Fe LDHs

### 2.1. Crystal Chemistry and Compositional Considerations of M(II)-Fe(III) LDHs

A commonly cited compositional range for hydrotalcite-like LDHs is x = 0.20–0.33, corresponding approximately to M(II)/Fe(III) molar ratios from 4:1 to 2:1 [36]. Within this interval, the layer charge is usually sufficient to stabilize the interlayer anion arrangement without introducing an excessive fraction of trivalent cations into the hydroxide sheets. For binary M(II)-Fe(III) LDHs, this range is a useful guideline rather than a universal single-phase stability field [48].

Its applicability depends strongly on the divalent metal. Ni-Fe, Mg-Fe, and Zn-Fe LDHs are commonly reported within the conventional x = 0.20–0.33 interval, although their phase purity still depends on synthesis conditions and post-synthesis treatment [49,50,51,52]. CoFe-LDHs are also frequently synthesized within this interval, including at a nominal Co(II)/Fe(III) ratio of 2:1. Their composition is more difficult to interpret when Co(III) is present or forms during the reaction. Co oxidation may increase the effective layer charge if Co(III) remains incorporated in the hydroxide sheets or may instead accompany reconstruction into CoFe oxyhydroxides. These possibilities should be distinguished from the charge and composition of the original LDH lattice [53,54,55,56].

Mn-Fe and Cu-Fe systems require greater caution. In Mn-Fe materials, Mn oxidation state and structural location may change during synthesis, treatment, or application. Mn(II)-Fe(III) layered phases can form under controlled conditions, whereas oxidation may generate higher-valent Mn within the layers or produce separate MnOOH, birnessite-like, MnO_x_, or spinel phases. Mn(III) or Mn(IV) detection after reaction therefore does not demonstrate that these species were present in the pristine LDH or incorporated into its hydroxide sheets [10,52,57,58,59,60].

Cu(II) is susceptible to Jahn–Teller distortion in octahedral hydroxide coordination, producing locally anisotropic Cu-O bond environments and potentially promoting structural distortion or reduced long-range order in Cu-containing layered phases [61]. Differences in Cu(II) and Fe(III) hydrolysis can also favor copper hydroxynitrate, Cu(OH)_2_, CuO, or poorly crystalline Fe-rich products, particularly under Cu-rich conditions [62]. Cu incorporation consequently depends on composition, pH, precipitation route, and aging and should not be inferred from the nominal precursor ratio. Because conventional PXRD may not distinguish incorporated Cu from poorly crystalline Cu-rich secondary phases, local-structure methods such as Cu K-edge XAS or EPR are desirable [63].

Ca-Fe layered hydroxides and Fe(II)/Fe(III) green rust fall outside the conventional brucite-like M(II)-Fe(III) model. Ca-Fe phases are more appropriately interpreted as hydrocalumite- or AFm-related structures, whereas green rust is a mixed-valent Fe-only layered hydroxide in which the Fe(II)/Fe(III) ratio controls both layer charge and reducing capacity [2,42,64]. These systems are therefore discussed separately in Section 2.4.

In conventional brucite-like LDHs, cation incorporation is partly governed by compatibility, with six-coordinate sites in edge-sharing [M(OH)_6_] octahedral layers. The effective ionic radius of high-spin Fe(III) is 0.645 Å, compared with 0.690 Å for Ni(II), 0.720 Å for Mg(II), 0.730 Å for Cu(II), 0.740 Å for Zn(II), 0.745 Å for high-spin Co(II), and 0.830 Å for high-spin Mn(II) [64,65]. These differences are generally compatible with the formation of a common hydroxide layer, although the larger Mn(II)-Fe(III) mismatch may require greater accommodation of unequal metal–OH bond lengths.

Ionic radius compatibility alone does not establish LDH stability [49]. Cu(II) is an important counterexample because acceptable size matching does not eliminate Jahn–Teller distortion, unequal hydrolysis behavior, or competition from Cu-containing secondary phases. More generally, phase formation is controlled by the combined effects of metal hydrolysis, precipitation kinetics, oxidation state, ligand coordination, pH, supersaturation, aging, and interlayer chemistry. Redox-active Co and Mn systems may also change composition after synthesis or during application, so the structure of the reacted material should not be inferred directly from the pristine precursor ratio [38,61,62].

Ca-Fe layered hydroxides and Fe(II)/Fe(III) green rust illustrate the limits of applying the same ionic radius argument to all Fe-containing layered hydroxides. Even for six-coordinate Ca(II), the effective ionic radius is approximately 1.00 Å, substantially larger than that of high-spin Fe(III); moreover, Ca(II) commonly accommodates coordination numbers greater than six. This supports the interpretation of Ca-Fe layered hydroxides as hydrocalumite- or AFm-related phases rather than ordinary brucite-like M(II)-Fe(III) solid solutions [42,66]. In green rust, high-spin Fe(II) and Fe(III) have six-coordinate radii of approximately 0.780 and 0.645 Å, respectively; this mismatch is compatible with an edge-sharing Fe-hydroxide layer, but phase stability is governed mainly by redox potential, pH, Fe(II)/Fe(III) ratio, oxygen exposure, and interlayer anion identity [2,67].

Here, the term “brucite-like” describes the topology of the edge-sharing octahedral hydroxide layers and does not imply that crystalline brucite is a required precursor during synthesis. Pyroaurite is the natural Mg-Fe(III) carbonate member of the hydrotalcite supergroup and should not be used as a universal structural designation for all M(II)-Fe(III) LDH systems [64,68].

Layer charge is another central crystal chemical parameter. As x increases, the positive charge density of the hydroxide layer grows, and a larger amount of interlayer anions is required to compensate for the charge [68]. This affects interlayer hydration, electrostatic layer–anion interactions, basal spacing, anion exchange, swelling, delamination, and reconstruction. The measured basal spacing consequently reflects not only layer composition but also the identity, orientation, and hydration of interlayer species. Shifts in basal reflections may indicate anion exchange or hydration changes, but they do not independently prove successful intercalation because stacking disorder, mixed phases, variable water content, and changes in crystallinity can produce similar diffraction effects [68,69,70].

The nominal precursor ratio must be distinguished from the actual composition of the solid. Selective precipitation, incomplete incorporation of one metal, dissolution during washing, oxidation, and secondary-phase formation may all change the final M/Fe ratio [37]. Therefore, quantitative elemental analysis by ICP-OES, ICP-MS, XRF, or another suitable method is needed to verify bulk composition. Even then, alignment between the measured bulk M/Fe ratio and the nominal LDH stoichiometry does not demonstrate that both metals occupy the same layered phase, because an apparently appropriate bulk ratio may also arise from mixtures containing poorly crystalline or amorphous secondary components. It should be interpreted together with X-ray diffraction, vibrational spectroscopy, microscopy, and, where possible, local-structure-sensitive characterization [38].

The spatial distribution of Fe within the layers also cannot usually be established by conventional PXRD. Although long-range cation ordering may sometimes produce superstructure reflections, average diffraction patterns generally cannot distinguish random mixing from short-range ordering or nanoscale Fe-rich clustering in poorly crystalline LDHs. EXAFS and total-scattering pair distribution function analysis can probe local coordination and metal–metal distances, while XANES provides information on oxidation state and local symmetry. Mössbauer spectroscopy, supported where relevant by magnetic measurements, can distinguish Fe oxidation states and local hyperfine environments and may provide indirect evidence of magnetic or electronic interactions among Fe sites. Neutron total scattering, solid-state NMR for suitable nuclei, and atomically resolved STEM-EELS or STEM-EDS may provide additional evidence of cation ordering and spatial heterogeneity [49,68,71].

Local Fe-Fe and M-Fe configurations can alter magnetic and electronic coupling, as demonstrated for NiFe and related LDHs. Their effects on adsorption site distribution and environmental catalytic activity remain less systematically established [72,73]. Random cation distribution should therefore be treated as a simplifying structural model rather than an experimentally demonstrated feature unless supported by local-structure evidence.

The crystal chemistry of binary M(II)-Fe(III) LDHs is governed by Fe(III) fraction, layer charge, geometric compatibility, hydrolysis and precipitation behavior, oxidation state, interlayer chemistry, and local cation distribution. The conventional x = 0.20–0.33 range provides a useful starting point, but actual composition, phase purity, and structural homogeneity must be verified for each M-Fe system (Table 1).

### 2.2. Fe Hydrolysis, Oxidation State, and Redox Transformation

Iron controls both the layer charge and the chemical behavior of binary Fe-containing LDHs. In conventional M(II)-Fe(III) LDHs, Fe(III) occupies predominantly octahedral sites within the hydroxide layers and generates a net positive layer charge that is compensated by interlayer anions. Surface Fe-OH groups may also contribute to the uptake of oxyanions such as phosphate, arsenate, chromate, molybdate, and vanadate through electrostatic interactions, ligand exchange, and surface complexation, depending on solution pH and anion identity. In redox-active materials, Fe may additionally participate in electron transfer, oxidant activation, and application-induced structural reconstruction [35,38].

More importantly for synthesis, Fe(III) strongly tends to hydrolyze in aqueous solution. Depending on pH, concentration, complexing species, temperature, and mixing conditions, Fe(III) may form hydrolyzed monomers, oligomeric species, poorly crystalline Fe(III) hydroxides, or oxyhydroxides. During rapid or spatially non-uniform alkalization, Fe-rich species may precipitate before the second metal is fully incorporated into the developing solid. This can result in compositional gradients, Fe-rich domains, or secondary iron hydroxide and oxyhydroxide phases [37].

Consequently, the formation of a homogeneous M(II)-Fe(III) LDH cannot be predicted from ionic radius compatibility alone. It also depends on the relative hydrolysis and solubility behavior of both metals, their coordination preferences and redox stability, the pH trajectory, local supersaturation, interlayer anion identity, complexation, oxidation during precipitation, and subsequent aging. These factors provide the chemical basis for the alternative nucleation and phase-transformation pathways discussed in Section 2.3.

In Co-Fe, Ni-Fe, Mg-Fe, Zn-Fe, Mn-Fe, Cu-Fe, and Ca-Fe layered hydroxides synthesized from Fe(III) salts, iron should generally be assumed to remain predominantly in the Fe(III) state unless mixed valence, chemical reduction, or a redox transformation is directly demonstrated. The assignment of structural Fe(II) therefore requires caution. In particular, a minor low-binding-energy component in an Fe 2p XPS spectrum should not, by itself, be considered definitive evidence of Fe(II), especially when the synthesis was conducted without reducing agents or oxygen-excluding conditions [74].

Determining Fe speciation from XPS alone is difficult because the Fe 2p region of high-spin iron compounds contains strongly overlapping multiplet, spin-orbit, and satellite features. Simplified fitting, in which one component is assigned to Fe(II) and another to Fe(III), may generate artificial Fe(II)/Fe(III) ratios when the multiplet structure and satellite contributions are not represented appropriately [75]. XPS probes mainly the near-surface region and may therefore not represent the oxidation state or composition of the bulk material [76].

A more reliable evaluation of Fe speciation should combine surface-sensitive XPS with complementary bulk-sensitive methods. ^57^Fe Mössbauer spectroscopy and Fe K-edge XANES provide more robust bulk-sensitive constraints on Fe oxidation state and local Fe environments, whereas EXAFS is primarily useful for determining local coordination environments. Wet-chemical redox titration may provide quantitative Fe(II)/Fe(III) ratios when sampling and analysis are performed under oxygen-free conditions. Magnetic measurements can provide additional supporting evidence, where relevant, but rarely establish Fe oxidation state unambiguously when used alone [77]. XPS should therefore be described primarily as evidence of near-surface Fe speciation rather than as a standalone measurement of bulk Fe oxidation state [78].

This distinction is especially important for Fe(II)-containing systems such as green rust. Because structural Fe(II) is readily oxidized by air, green rust samples require oxygen-free synthesis, washing, storage, and inert transfer whenever Fe speciation is evaluated. Minimizing air exposure and comparing ex situ results with in situ or operando data are important for distinguishing genuine reaction-induced oxidation from artifacts introduced during handling or sample preparation.

The redox behavior of Fe depends on the identity of the second metal and on the reaction conditions. Electronic coupling through M-O(H)-Fe environments has been demonstrated most clearly for NiFe- and CoFe-based electrocatalysts under oxygen-evolution conditions, where operando studies revealed coupled structural and electronic changes [72]. In MnFe- and CuFe-based oxidant-activation systems, system-specific studies also indicate coupled or defect-mediated electron-transfer pathways, although comparable operando evidence remains more limited [10,29,59]. Such interactions may influence oxidant adsorption, electron transfer, active-species formation, and surface reconstruction, but these effects should not be assumed to occur identically across all M-Fe systems. Claims of specific metal redox cycles should, where possible, be supported by complementary evidence such as in situ/operando spectroscopy, bulk-sensitive oxidation-state analysis, and solution-phase metal balances, rather than by ex situ XPS alone. Quenching and selective-probe experiments may help identify reactive solution or surface species, but they do not independently establish the oxidation-state cycle of the metal centers. Small fitted components or minor binding-energy shifts in ex situ XPS spectra should be treated as tentative rather than definitive evidence of catalytic redox cycling [75].

During operation, Mn-Fe, Co-Fe, and NiFe layered materials may undergo oxidation-state and coordination changes that alter the identity of the active phase. Oxidative conditions can convert Mn(II) or Co(II) into higher-valent hydroxide or oxyhydroxide species, while NiFe-LDH may reconstruct under anodic polarization into a Ni-Fe oxyhydroxide-like phase [10,37,72]. Green rust is especially sensitive to oxidation because the consumption of structural Fe(II) directly changes its reducing capacity and promotes transformation into Fe(III)-rich oxides or oxyhydroxides [67]. Post-reaction oxidation states and phase composition should therefore be characterized rather than inferred from the pristine material.

### 2.3. Intermediate Phases and Phase-Formation Pathways

Geometric compatibility between M(II) and Fe(III) within an octahedral hydroxide layer does not determine how the LDH phase actually forms. Coprecipitation is commonly represented as the simultaneous incorporation of both metals into a homogeneous layered solid, but this description is only a simplified model. In practice, Fe-containing systems may form through sequential hydrolysis, poorly ordered hydroxide or oxyhydroxide precursors, adsorption of the second metal, dissolution–reprecipitation, and structural reorganization [46].

Because Fe(III) may hydrolyze and precipitate before the divalent metal, the first solid formed during alkalization is not necessarily the final LDH. The extent to which this occurs depends on the relative hydrolysis and solubility of the two metals, the rate of base addition, local mixing, pH evolution, supersaturation, complexation, redox conditions, temperature, and aging time. Direct time-resolved evidence for formation pathways remains concentrated in CoFe, NiFe, and green rust systems, whereas MgFe, ZnFe, MnFe, CuFe, and CaFe are still commonly interpreted from the ex situ characterization of final products.

A clear example has been reported for Co-Fe systems prepared under high-supersaturation/variable-pH coprecipitation conditions. pH-resolved characterization showed that ferrihydrite can appear as an early solid phase. Cobalt may then adsorb onto this Fe-rich precursor, followed by hydrolysis, redox interactions, dissolution, and reprecipitation, leading to the final CoFe-LDH structure [37]. In this case, the first precipitate is not the final LDH, but a temporary precursor involved in the phase transformation. During homogeneous precipitation with triethanolamine and urea, an Fe(III)-rich oxyhydroxide intermediate formed first, while Ni(II) remained complexed in solution. Gradual alkalization during urea decomposition then promoted the transformation of these early species into well-crystallized hexagonal NiFe-LDH platelets, showing that the initial precipitate was not the final phase [79].

A related pathway has been observed in mixed-valent Fe systems, where time-resolved synchrotron diffraction showed that ferrihydrite can transform into Fe(II)-Fe(III) green rust under reducing conditions [2,39]. This finding supports the broader possibility that poorly crystalline Fe-rich precursors can participate in the formation of layered Fe-containing hydroxides. However, it does not imply that ferrihydrite is an obligatory intermediate in all M-Fe LDH systems.

The identity, abundance, and lifetime of intermediate phases are strongly system-dependent. In some metal combinations, direct formation of a mixed M(II)-Fe(III) layered precursor or rapid near-simultaneous incorporation of both metals may dominate. In others, amorphous or poorly ordered precursors may transform so rapidly that they are difficult to detect using conventional ex situ characterization. Oxidation during precipitation, aging, washing, or drying may further alter the pathway, particularly in Mn-, Co-, and Fe(II)-containing systems. Interlayer anions can also affect nucleation and phase stability through differences in charge, hydration, coordination ability, and affinity for the developing hydroxide layers [46].

Studies of M-Fe LDH formation should therefore distinguish between the initial precipitate, transient intermediates, the aged product, and the phase present under application conditions. Time-resolved or pH-resolved characterization is particularly valuable because analysis of only the final dried material cannot reveal whether the LDH formed directly or through precursor transformation. Recognizing these alternative pathways helps explain why nominally similar coprecipitation procedures may produce materials with different crystallinity, metal distributions, phase purity, morphology, redox behavior, and environmental performance.

Figure 2 illustrates direct coprecipitation, precursor-mediated transformation, dissolution–reprecipitation, structural reorganization, and redox-assisted pathways. These mechanisms are not necessarily mutually exclusive and may occur concurrently or sequentially depending on the metal system and synthesis conditions. In the schematic, blue circles represent M(II) species, orange circles represent Fe(III) species, and gray circles represent OH^−^. Cloud-like shapes denote poorly ordered or precursor hydroxide phases, whereas stacked platelets represent layered products. Solid arrows indicate the proposed direction of phase evolution, while dashed arrows in panel C indicate exchange, dissolution, or redistribution of species during structural reorganization. The colors used for panels A–D serve only to distinguish the four conceptual pathways and do not represent specific chemical species.

### 2.4. Conventional M(II)-Fe(III) LDHs, Ca-Fe Layered Hydroxides, and Fe(II)/Fe(III) Green Rust

The Fe-containing layered materials considered in this review comprise three structurally distinct groups whose principal characteristics have been introduced in Section 1.3, Section 2.1 and Section 2.2. Their main differences and mechanistic implications are summarized in Table 2.

These distinctions determine how to interpret structural evolution under operating conditions. Conventional M(II)-Fe(III) LDHs may remain layered or reconstruct into new active phases, Ca-Fe layered hydroxides undergo coupled dissolution–precipitation, whereas green rust consumes structural Fe(II) during reductive transformations [72,81,82]. Structural comparisons should therefore distinguish the initial layered material, the phase present under operation, and the final recovered or transformed products.

## 3. Precipitation Parameters and Post-Synthetic Control of Structure and Morphology

The precipitation reactor integrates a set of coupled synthesis variables, including the pH trajectory, feed rate, metal concentration, mixing, temperature, atmosphere, complexants, interlayer anion, and aging conditions. Together, these parameters control local supersaturation, metal hydrolysis, nucleation and growth, intermediate-phase formation, final composition, and the stacking and aggregation of the resulting particles (Figure 3). The sequence represents coupled relationships rather than a strictly linear reaction pathway. The colored diffraction profiles are schematic and illustrate possible differences in peak position, intensity, and broadening; the colors do not correspond to specific M-Fe systems or samples. Colored circles schematically represent different dissolved or precipitating chemical species, while clustered circles indicate an early precipitate or aggregate. Stacked platelets represent the development of a layered hydroxide product. Arrows indicate the conceptual direction of influence or material evolution and should not be interpreted as a strictly linear reaction sequence.

### 3.1. pH, Local Supersaturation, and Precipitation Sequence

pH is one of the principal variables controlling the synthesis of binary M-Fe layered hydroxides. It affects metal hydrolysis, nucleation, hydroxide layer formation, and the incorporation or competition of charge-compensating interlayer anions. Conventional M(II)-Fe(III) LDHs are commonly precipitated under mildly to strongly alkaline conditions, often around pH 9–11, although the optimum range depends on the metal pair, precipitation mode, interlayer anion, and complexing environment [50,57,83]. These conditions promote layer formation but do not necessarily prevent the transient precipitation of Fe-rich hydroxide or oxyhydroxide precursors. Where such intermediates form, pH control can influence their abundance, persistence, and subsequent transformation into the layered product. The required conditions also differ among structural classes: hydrocalumite-like Ca-Fe layered hydroxides generally form under more strongly alkaline conditions, often near pH 13, whereas Fe(II)/Fe(III) green rust additionally requires redox control and the exclusion of oxygen [2,84,85].

Supersaturation controls the balance between nucleation and crystal growth but should be considered as a local rather than purely bulk solution parameter. Rapid reagent addition, concentrated alkaline feeds, or insufficient micromixing can create transient regions of high local supersaturation, promoting rapid precipitation and a high density of nuclei. Depending on the M-Fe composition, mixing conditions, interlayer anions, and subsequent aging, the resulting products may exhibit smaller coherent-scattering domains, irregular aggregation, less ordered layer stacking, or secondary-phase formation. In Fe-containing systems, Fe-rich hydroxide or oxyhydroxide intermediates may also form, although their occurrence and transformation are strongly dependent on pH trajectory, metal speciation, and complexation conditions [79,86].

These features should not be equated automatically with beneficial defects or ultrathin morphology. High local supersaturation does not necessarily produce accessible edge sites, vacancies, or enhanced catalytic activity, and broad diffraction features should not be used alone to infer defect density or nanosheet thickness. Such assignments require independent microscopic, spectroscopic, or detailed diffraction evidence [87,88,89].

Lower, more controlled supersaturation generally favors slower nucleation and more extended crystal growth, particularly when the nucleation and aging stages are separated [18]. In NiFe-LDH, homogeneous precipitation through gradual pH increase during urea decomposition has produced highly crystalline hexagonal platelets with submicrometer-to-micrometer lateral dimensions and relatively uniform thickness [79]. In a template-assisted synthesis of mixed-valent Co-Fe LDH hollow spheres, slow NaOH addition produced flower-like shells of relatively large, mainly edge-on platelets, whereas rapid addition increased local supersaturation and formed raspberry-like shells composed of smaller, predominantly face-on nanoparticles. Thus, the base-addition rate can affect particle size, platelet orientation, and hierarchical morphology. However, this trend should not be generalized to conventional Co(II)-Fe(III) LDHs because it was observed in a templated mixed-valent system [90,91]. This result demonstrates that reagent-delivery kinetics can strongly affect particle organization, but it should not be generalized directly to conventional Co(II)-Fe(III) LDHs because template effects, mixed valence, and system-specific precipitation chemistry were also involved. Similarly, ultrathin CoFe-LDH nanosheets, reported to contain high defect densities, have been obtained under selected synthesis conditions, but rapid base addition should not be regarded as a general route to defect-rich nanosheets unless the relationship is independently demonstrated for the investigated system [92].

The sequence and mode of reagent addition further influence phase development by determining the spatial and temporal overlap between metal hydrolysis and hydroxide availability. The rapid addition of a base to a mixed-metal solution may generate local high-pH zones in which Fe(III) precipitates before the complete incorporation of the divalent metal, producing Fe-rich intermediates or impurities [46]. Co-titration of metal and alkaline solutions under constant-pH conditions can reduce abrupt local changes in pH and supersaturation, thereby improving phase control and synthesis reproducibility [41,93]. Constant-pH operation does not, however, guarantee homogeneous cation incorporation, which must still be verified through compositional and phase-sensitive characterization.

Comparative ZnFe-LDH studies have shown that constant-pH coprecipitation can yield higher dye uptake than direct or variable-pH procedures, although this improvement cannot be attributed to crystallinity alone, as surface area, aggregation, composition, and site accessibility may also change [86]. In MnFe-LDH, constant-pH low-supersaturation coprecipitation successfully produced the layered phase, while variations in crystal growth and agglomeration were primarily associated with the interlayer anion rather than with systematic changes in synthesis pH [57]. These observations emphasize that nominal pH is insufficient as a transferable synthesis descriptor unless reagent delivery, mixing, supersaturation history, and interlayer chemistry are also considered.

The relationship between structural order and environmental performance is not binary. Greater structural coherence may improve phase stability and reproducibility, whereas smaller coherent domains or thinner platelets may increase the geometric fraction of edge and termination environments. Their actual accessibility, however, depends on aggregation, restacking, porosity, surface chemistry, and the material state under operating conditions. Well-characterized disorder or defect populations may further modify local coordination environments, but such structural features do not constitute direct evidence of reactive sites. Excessive disorder can instead promote aggregation, dissolution, metal leaching, or transformation into poorly defined phases, and enhanced activity may originate from secondary phases or reaction-generated structures rather than from defects in the pristine LDH used [94,95]. The relevant degree of structural order is consequently system- and mechanism-dependent and should be evaluated together with phase identity, site accessibility, and structural evolution under operating conditions.

### 3.2. Metal Ratio, Precursor Concentration, and Reagent Addition Mode

As discussed in Section 2.1, the commonly targeted Fe(III) fraction in conventional M(II)-Fe(III) LDHs is x = 0.20–0.33. In coprecipitation, this ratio is important because it controls layer charge, Fe content, interlayer anion demand, and the probability of secondary Fe-rich phases. The same nominal M/Fe ratio may produce different phase purity and morphology depending on the metal pair, because each system has different hydrolysis, redox, and structural constraints [53,96]. The precursor ratio should therefore be regarded as a synthesis input rather than a direct descriptor of the composition of the resulting layered phase.

The incorporated Fe(III) fraction also affects morphology and local structure. A higher Fe(III) fraction increases the number of Fe-containing sites and the positive layer charge, thereby increasing the demand for charge-compensating interlayer anions and strengthening electrostatic layer–anion interactions. This can modify interlayer hydration, stacking, and crystallinity. However, excessive Fe incorporation can promote Fe-rich local environments, short-range ordering, or separate Fe-containing oxide/oxyhydroxide phases, depending on the metal pair and synthesis conditions. In some systems, local Fe enrichment or Fe-Fe nearest-neighbor formation may modify redox, adsorption, or catalytic activity, but these effects are not universally beneficial and can also reduce structural homogeneity or phase stability. Therefore, the optimal metal ratio should be selected based on both phase stability and the target function [73,96]. Selection of the M/Fe ratio should therefore consider measured solid composition, phase stability, local cation distribution, and the reactive-site environment relevant to the intended application rather than nominal stoichiometry alone.

Precursor concentration can influence supersaturation, nucleation, and particle growth, but its effect cannot be separated from local OH^−^ concentration, mixing time, reagent addition rate, metal speciation, and saturation with respect to both the LDH phase and competing Fe-rich precipitates. Under otherwise comparable mixing and feed conditions, higher metal concentrations may promote more abrupt local supersaturation, rapid precipitation, aggregation, or the formation of poorly ordered and secondary phases [47,97]. Dilution can reduce such gradients but does not necessarily produce larger, less defective, or better-dispersed platelets because nucleation density, heterogeneous nucleation, reaction time, aging, and drying-induced aggregation may also change. Precursor concentration should therefore not be interpreted as an independent morphology-control parameter.

Additionally, this concentration dependence creates a process-level trade-off. Dilute synthesis reduces solid productivity and increases the volume requiring processing and washing. Continuous-flow, high-gravity, separate-nucleation-and-aging, and related intensified approaches can potentially decouple part of this trade-off by improving control of reagent contact and mixing at higher throughputs, although improved phase purity or morphology should be demonstrated experimentally rather than inferred from reactor configuration alone [30,47,98].

The concentration and delivery mode of the alkaline reagent can influence precipitation by affecting the rate and spatial uniformity of OH^−^ supply. When micromixing is insufficient, concentrated alkaline feeds are expected to increase local pH and supersaturation gradients near the addition point. Studies on LDH coprecipitation have shown that precursor concentration, solution addition rate, and stirring rate can affect particle size and particle-size distribution, although direct quantitative evidence for such local gradients in binary M-Fe LDH synthesis remains limited [79,99]. Consequently, mechanistic explanations based on presumed local pH or supersaturation gradients should be distinguished from gradients that have been directly measured or otherwise experimentally constrained.

For NiFe-LDH, homogeneous precipitation using urea and a metal-complexing agent enabled a gradual increase in pH and controlled release of Ni^2+^ and Fe^3+^, producing phase-pure, highly crystalline platelets. However, gradual, or homogeneous OH^−^ generation does not necessarily produce a more ordered material. An epoxide-driven homogeneous alkalinization route yielded a low-dimensional NiFe-LDH with nanosized crystalline domains, pronounced Fe clustering, and a defect-rich structure. Fe-rich hydroxide or oxyhydroxide intermediates were also detected during both formation pathways [79,100]. Fe-rich hydroxide or oxyhydroxide intermediates were detected during both formation pathways. These contrasting results illustrate that the structural outcome of homogeneous precipitation depends not simply on the rate of alkalinization but also on metal complexation, precursor speciation, transformation kinetics, and aging.

The reagent-addition mode determines how metal ions, hydroxide ions, and interlayer anions interact during precipitation. Direct base addition is experimentally simple but can create transient pH and supersaturation gradients when reagent delivery outpaces local mixing. Variable-pH precipitation exposes the system to successive hydrolysis and supersaturation regimes. This may increase phase or compositional heterogeneity under some conditions, but it also provides valuable information on precipitation plateaus, intermediate phases, and LDH formation pathways. In CoFe systems, variable-pH studies revealed the initial precipitation of a ferrihydrite-like Fe-rich phase, subsequent Co(II) sorption and coupled Co-Fe redox reactions, and its gradual transformation into a layered hydroxide phase. Variable-pH precipitation can therefore serve both as a preparative route and as a diagnostic method for studying phase evolution [37,53]. Variable-pH precipitation can therefore function both as a preparative method and as a means of resolving phase-evolution pathways.

Constant-pH coprecipitation, particularly through the controlled co-titration of the metal and alkaline solutions, restricts precipitation to a selected pH range and facilitates the control of synthesis conditions. It is therefore often preferred when reducing uncontrolled sequential precipitation and improving reproducibility are priorities. However, the homogeneous incorporation of Fe and the second metal should not be assumed based on pH control alone and requires confirmation through bulk and local compositional analyses [86,93,98]. Constant-pH operation should therefore be regarded as a method for controlling precipitation history rather than as evidence of compositional homogeneity.

Taken together, the M/Fe ratio, precursor concentration, and reagent-addition mode are coupled rather than independent synthesis variables. Their effects converge through metal speciation, local supersaturation, nucleation and transformation kinetics, and the temporal sequence of cation incorporation. Consequently, similar nominal recipes can yield structurally different products, whereas different precipitation strategies may converge toward similar layered phases after aging. Therefore, optimization should prioritize reproducible phase identity, measured composition, structural homogeneity, and accessible reactive-site environments rather than a single synthesis parameter.

### 3.3. Temperature, Aging, Mixing, and Atmosphere

Temperature affects the kinetics of M-Fe LDH formation and the structural evolution of the precipitate during aging. Moderate heating can accelerate hydrolysis, precursor dissolution, ion redistribution, and recrystallization, but its effect depends strongly on metal speciation, complexation, pH evolution, and aging time. In a homogeneous NiFe-LDH synthesis, heating to 100 °C promoted the gradual conversion of an Fe–oxyhydroxide/Ni-complex precursor into phase-pure, highly crystalline NiFe-LDH platelets. Intermediate samples collected during heating contained both developing platelets and residual poorly crystalline Fe-rich material, which was progressively consumed during prolonged treatment [79,101,102,103]. This example illustrates that heating can facilitate precursor-to-LDH transformation rather than simply increasing the crystallinity of an already formed layered phase.

Post-precipitation thermal treatment can increase the structural order of NiFe-LDHs. In an acid-assisted etching–recrystallization study, hydrothermal treatment at 100 °C increased structural coherence, with the in-plane coherent-scattering domain length reaching 22.7 nm for NiFe ER5, compared with 12.4 nm for the pristine NiFe control and 10.8 nm for the etched NiFe E5 sample [104]. However, the treatment also generated 5–20 nm Fe_3_O_4_ nanoparticles, whose abundance and size increased with nitric acid loading and hydrothermal temperature. Therefore, sharper LDH reflections indicate increased coherence in the remaining layered phase but do not, by themselves, demonstrate improved overall phase purity [105]. Sharper LDH reflections, or larger coherent-scattering domains, therefore indicate increased structural coherence in the remaining layered phase but cannot, by themselves, demonstrate increased phase purity in the bulk material.

Temperature and aging should consequently be treated as coupled kinetic variables rather than as universally beneficial routes to greater structural order. Their effects depend on pH, alkalinity, reagent addition, precursor concentration, mixing, and the chemical stability of the targeted layered phase. Moderate heating may promote crystallization and particle growth, whereas prolonged treatment under strongly alkaline conditions can destabilize the layered structure. For example, exposure of NiFe-LDH to concentrated KOH at 80 °C resulted in its transformation into β-Ni(OH)_2_ and FeOOH-containing phases. Therefore, temperature and aging should be treated as coupled kinetic variables rather than as universally beneficial methods for increasing crystallinity [106].

Studies of isolated ferrihydrite provide useful mechanistic context for Fe-rich intermediates identified during M-Fe layered hydroxide synthesis, particularly regarding dissolution–reprecipitation and transformation under alkaline or carbonate-containing conditions [107,108]. Such pathways should not be transferred directly to binary M-Fe suspensions. The second metal, interlayer anions, complexing agents, and the developing layered phase can modify Fe speciation, solubility, interfacial processes, and transformation kinetics. Ferrihydrite model systems therefore provide mechanistic hypotheses rather than direct evidence for the pathway operating in a specific M-Fe synthesis.

Aging begins after initial precipitation but can remain an active stage of phase formation rather than merely a period of crystal growth. Depending on the system, it can promote dissolution–recrystallization, particle growth, improved stacking order, interlayer organization, and cation redistribution within or between hydroxide phases [102,108]. These processes often sharpen basal XRD reflections and increase structural coherence. In metastable systems, prolonged aging under strongly alkaline, oxidizing, carbonate-rich, or hydrothermal conditions may also promote phase transformation or the formation of secondary oxide/oxyhydroxide products, especially when Fe-rich poorly crystalline intermediates are present [103,109,110,111]. Consequently, the structure recovered after aging may reflect continued phase evolution rather than the simple maturation of the initially precipitated LDH.

Mixing controls the spatial and temporal distribution of metal ions, OH^−^, and interlayer anions during precipitation. Insufficient micromixing can create local pH and supersaturation gradients near the feed point, causing non-uniform nucleation and aggregation. More uniform OH^−^ delivery may reduce localized Fe(III) hydrolysis, although suppression of Fe-rich intermediates requires direct phase and speciation evidence [98,102,108].

Mixing should therefore be described using engineering parameters, including impeller type and diameter, vessel geometry, liquid volume, baffles, stirring rate, feed position, addition rate, and mixing time. Reporting rpm alone is insufficient because identical rotational speeds do not produce equivalent hydrodynamic conditions across different reactor scales and geometries. Gabriel et al. showed that stirring rate, aging time, and baffles affected LDH yield and structural characteristics [108]. In continuous MgFe-LDH synthesis, mixer geometry, flow rate, inlet configuration, and sheath flow influenced operability, particle deposition, and clogging [112]. Continuous-flow systems can improve control over reagent contact and residence time, but they do not automatically produce more uniform or higher-quality LDHs [113]. These observations highlight reactor design and mixing history as synthesis descriptors rather than secondary experimental details.

Atmosphere control serves different purposes depending on the M-Fe system and the target interlayer composition. For nitrate- or chloride-intercalated LDHs, exposure to atmospheric CO_2_ can introduce carbonate or produce carbonate-containing phases [114,115]. This effect is especially relevant for CaFe hydrocalumite-like materials because Ca^2+^ readily reacts with carbonate. CaFe-Cl LDHs have therefore been synthesized under N_2_ during precipitation and aging to limit carbonate contamination [42,115,116].

Redox control is a separate consideration. In MnFe systems, the required atmosphere depends on the targeted Mn and Fe oxidation states. Reduced Mn(II)- or Fe(II)-containing compositions may undergo dissolution or transformation if oxidation is not controlled, whereas deliberate oxidation of Mn(II) to Mn(IV) can stabilize some MnFe-LDH structures but may also produce associated birnessite-like domains. Atmosphere requirements should therefore be defined for the intended Mn/Fe valence composition rather than applied uniformly to all MnFe-LDHs [58]. Strict anoxic handling is essential for Fe^II^/Fe^III^ green rust. Oxygen must be excluded during synthesis, washing, storage, transport, and characterization because even limited exposure can oxidize Fe^II^ and generate secondary ferric phases [2,85].

### 3.4. Interlayer Anion Chemistry and Carbonate Control

In Fe-containing LDHs, carbonate is not only an interfering or unintentionally incorporated anion but also an important structural component. Its divalent charge, favorable geometry, and strong electrostatic and hydrogen-bonding interactions often promote ordered layer stacking, phase reproducibility, and resistance to dissolution. Carbonate-containing forms may therefore be advantageous when structural stability is more important than extensive interlayer exchange. Carbonate can also influence metal hydrolysis, precipitation pathways, and reconstruction behavior during synthesis or rehydration.

Strong carbonate retention reduces interlayer lability and can compete with exchangeable interlayer anions or dissolved oxyanions. Studies of Mg-Fe and other Fe-containing LDHs used for nitrate or phosphate removal show that carbonate contamination or competition can reduce anion-exchange capacity, with carbonate frequently acting as one of the strongest interfering anions in nitrate removal [11,20,117]. This effect is nevertheless mechanism-dependent. Phosphate and other oxyanions may still be removed through surface complexation, ligand exchange, precipitation, or structural transformation. Carbonate should therefore be regarded as a factor that restricts interlayer tunability rather than as an absolute barrier to adsorption.

The carbonate’s effect consequently depends on the dominant removal mechanism. It may be unfavorable when rapid interlayer exchange, delamination, or access to a highly labile interlayer is required. In contrast, carbonate-containing LDHs may remain suitable when adsorption is dominated by external-surface complexation or when phase stability, low dissolution, and repeated operation are priorities. Carbonate incorporation or release can also influence phase reconstruction during calcination–rehydration cycles, meaning that the interlayer composition of the recovered material should not automatically be assumed to reproduce that of the precursor.

Noncarbonate interlayers such as nitrate and chloride generally provide greater chemical tunability. Nitrate-intercalated NiFe-LDHs, for example, can exhibit enlarged interlayer spacing and facilitate exfoliation into monolayers, whereas Mg-Fe-Cl LDHs have been shown to exchange interlayer chloride with phosphate during water treatment [19,69,118,119]. Sulfate behaves differently because its divalent charge and hydration environment can modify interlayer electrostatics, water organization, hydrogen bonding, and layer spacing. In NiFe systems, sulfate-containing interlayers have been associated with ultrathin nanosheet formation, although inorganic sulfate may be displaced by carbonate under alkaline conditions. More strongly retained organosulfates, including dodecyl sulfate, can produce larger interlayer separations and greater resistance to carbonate exchange [19,120,121]. These comparisons demonstrate that interlayer anion identity affects not only exchangeability but also hydration, stacking, exfoliation behavior, and the persistence of the targeted interlayer structure.

Carbonate incorporation may be intentional, through the addition of Na_2_CO_3_, or unintentional, through dissolved CO_2_, atmospheric exposure, reagent impurities, or water composition [114]. Even when nitrate or chloride metal salts are used, the resulting LDH may incorporate carbonate due to its strong affinity for positively charged LDH layers [117,119]. Therefore, nominal precursor anion identity should not be treated as evidence of final interlayer composition.

Preparation of carbonate-poor materials may require decarbonated water, carbonate-free bases, freshly prepared solutions, inert gas purging, sealed reactors, and protection from atmospheric CO_2_ during washing, storage, and drying [114,115]. In redox-sensitive systems, carbonate control may need to be combined with oxygen control. The required degree of atmospheric protection should nevertheless be determined by the experimental objective: stringent carbonate exclusion is particularly important when investigating anion exchange, exfoliation, or interlayer-specific chemistry, whereas it is less relevant when carbonate is intentionally selected as the interlayer anion.

Urea-assisted precipitation illustrates the coupling between precipitation chemistry and interlayer identity. Under typical synthesis conditions, gradual urea hydrolysis/decomposition generates alkalinity along with carbonate-containing species, thereby often favoring carbonate-containing interlayers. Modified urea routes can nevertheless produce nitrate- or chloride-containing products by controlling the precursor anion concentration, the urea-to-metal ratio, the reaction time, and the solution chemistry [122]. Urea precipitation should therefore not be classified simply as a “carbonate route”; rather, its interlayer outcome reflects the coupled evolution of pH, carbonate availability, precursor anion concentration, and crystallization kinetics.

Interlayer anion assignment requires complementary evidence. Basal spacing changes from XRD can support changes in interlayer organization but are insufficient to establish anion identity, as hydration state, stacking disorder, interstratification, and mixed interlayers can also affect basal reflections. Vibrational spectroscopy and elemental or solution analysis provide complementary chemical information. For example, carbonate-, chloride-, and sulfate-containing NiFe-LDHs have been evaluated using XRD, FTIR, and EDS as supporting elemental evidence, while nitrate-intercalated NiFe-LDHs with enlarged basal spacing were further supported by FTIR, XPS, TEM, and AFM. CaFe-LDH studies also show the usefulness of ion chromatography for tracking anion exchange in solution [19,84,118]. EDS can establish elemental presence but should not be treated as unambiguous evidence for the identity, location, or quantitative occupancy of an interlayer anion.

Interlayer anion chemistry is both a synthesis variable and a structural outcome. The anion introduced with the precursors may differ from that retained in the final material, particularly when carbonate is present during precipitation, washing, or storage. Consequently, interlayer composition should be experimentally verified and interpreted together with hydration state, basal spacing, and phase identity rather than inferred from the nominal synthesis recipe.

### 3.5. Additives, Complexing Agents, and Post-Synthesis Treatments

Urea, complexing agents, organic additives, and post-synthetic treatments provide additional control over M-Fe layered hydroxide formation by modifying alkalinization, metal speciation, nucleation, aggregation, and subsequent phase evolution. Their effects cannot, however, be separated from the accompanying changes in pH trajectory, interlayer chemistry, solvent composition, and aging conditions.

Urea is widely used for homogeneous or gradual alkalinization. During heating, its decomposition progressively increases solution alkalinity while also generating carbonate-containing species. Compared with the direct addition of a concentrated base, this approach can reduce abrupt local supersaturation and support more spatially uniform precipitation. In NiFe-LDH systems, urea-assisted synthesis has been shown to produce foam-like porous architectures composed of plate-like particles, whereas direct NaOH precipitation can yield micrometer-sized aggregates of irregular nanoparticles [123]. Such morphological differences should not be attributed to alkalinization rate alone because carbonate availability, metal speciation, aging, and solution composition also differ between the routes.

Complexing agents provide more direct control over metal speciation. Triethanolamine (TEA), for example, can delay metal hydrolysis and modify the relative availability of Ni and Fe during homogeneous NiFe-LDH formation. In combination with urea decomposition, complexation has been associated with Fe-rich precursor formation followed by gradual metal redistribution and recrystallization into well-ordered NiFe-LDH platelets [79,123,124,125]. Glycerol-containing media can also modify aggregation and particle organization, although their specific coordination or templating role remains less clearly established. In MnFe systems, EDTA-assisted synthesis under controlled atmosphere has produced EDTA-containing layered structures with expanded interlayer spacing and properties distinct from carbonate-containing analogues [57].

These examples demonstrate that additives should not be considered passive morphology modifiers. Strong ligands can change metal hydrolysis and precursor speciation, shift the temporal sequence of cation incorporation, alter the measured M/Fe ratio of the solid, and modify interlayer composition or phase identity [79]. Consequently, an additive-induced change in morphology cannot by itself demonstrate successful control of the original LDH structure. Phase identity, measured composition, interlayer chemistry, and metal speciation should be reassessed when complexing agents substantially modify the precipitation pathway.

Post-precipitation hydrothermal treatment provides another means of structural evolution. Under suitable conditions, moderate hydrothermal aging can increase coherent-domain size and improve layer stacking, but it may simultaneously promote particle growth, dissolution–reprecipitation, loss or redistribution of structural disorder, and secondary-phase formation [101,126]. The resulting increase in diffraction coherence should therefore not automatically be interpreted as an improvement of the material as a whole. As demonstrated for the NiFe systems discussed in Section 3.3, the increased structural coherence of the layered fraction can coexist with the formation of separate Fe-containing phases. Post-synthetic treatment should consequently be evaluated against both the structural state of the remaining LDH and the phase composition of the entire recovered solid.

Calcination represents a more fundamental transformation because it destroys the original layered structure and produces LDH-derived mixed oxides. Rehydration can partially restore layered structures through the memory effect, but the reconstructed material need not reproduce the composition, interlayer identity, porosity, cation distribution, or the reactive-site environment of the original LDH [84,127,128,129]. Materials subjected to calcination–rehydration should therefore be described as LDH-derived oxides, reconstructed layered phases, or LDO/LDH systems as appropriate, rather than being treated as structurally identical to the pristine precursor.

Additives and post-synthetic treatments should be interpreted as modifiers of the material’s formation and transformation pathway rather than as independent routes to a predetermined morphology. The same additive or treatment can affect metal speciation, interlayer chemistry, phase identity, particle organization, and structural coherence simultaneously. Establishing a causal relationship between a treatment and a specific structural feature therefore requires comparison against appropriate controls together with complementary phase, compositional, spectroscopic, and microscopic characterization.

Systematic parameter studies remain concentrated in NiFe-LDHs and CoFe-LDHs, whereas equivalent datasets are scarce for CuFe, MnFe, CaFe, and green rust systems (Table 3).

### 3.6. Green Chemistry and Resource Efficiency Considerations

Aqueous coprecipitation is often considered an environmentally favorable route for LDH synthesis because it avoids large quantities of organic solvents and can be performed using relatively simple equipment. However, the use of water as the reaction medium does not by itself make a synthesis “green”. A more complete assessment should consider reagent hazard and utilization, water and energy consumption, waste generation, auxiliary operations, product yield, and process productivity [33]. This is particularly relevant for LDHs because conventional coprecipitation may involve strongly alkaline conditions, extended aging, repeated washing and solid–liquid separation, and subsequent drying or thermal treatment.

Synthesis concentration and precursor choice introduce important trade-offs. Dilute precipitation may improve the control of local supersaturation but reduces solid productivity and increases the volume of the mother liquor and wash water generated per unit mass of product. Higher concentrations can improve volumetric productivity but may increase local pH gradients, aggregation, and secondary-phase formation. Soluble nitrate and chloride precursors are convenient, but their counterions remain in the liquid waste together with excess alkali. Precursor selection should therefore consider not only phase formation and interlayer chemistry but also salt loading, reagent purity, and downstream purification requirements [130].

Energy and utility requirements also depend on the synthesis route. Room-temperature or moderately heated coprecipitation generally requires less thermal input than hydrothermal aging, calcination, or repeated reconstruction treatments. Inert or anoxic conditions, required for green rust and some redox-sensitive systems, also increase gas consumption and process complexity [79]. A recent life-cycle assessment of a NiFe-LDH-based adsorbent identified electricity use and chemical inputs as major contributors to environmental impacts, further showing that sustainability should be assessed at the process level rather than inferred from the synthesis medium alone [131].

Accordingly, no LDH synthesis route should be described as “green” solely because it uses water or operates at relatively low temperatures. Sustainability claims should instead consider water and reagent demand, washing and separation steps, energy and gas consumption, waste stream composition, product yield, and solid productivity. Future studies should report these resource-use parameters alongside synthesis and structural data when discussing green chemistry or scalability [41].

## 4. Nanostructure, Morphology, and Reactive-Site Accessibility

### 4.1. Crystallinity, Basal Spacing, and Phase Purity

#### 4.1.1. Long-Range Order, Polytypes, and Peak Broadening

Crystallinity and phase composition are among the first criteria used to assess whether a layered hydroxide phase has formed. Conventional LDHs are commonly identified by basal (00l) reflections, such as (003) and (006) for rhombohedral polytypes, together with non-basal reflections related to in-plane ordering. Regular basal periodicity supports the presence of stacked hydroxide layers separated by hydrated interlayer anions, but an LDH-like pattern alone does not demonstrate a defect-free structure, a unique polytype, or complete phase purity.

Peak broadening should not be attributed automatically to small particle size. It may reflect limited coherent-domain size, microstrain, stacking faults, turbostratic disorder, interstratification, mixed hydration states, polytype intergrowths, or overlapping secondary phases [132,133]. These effects are structurally distinct. Stacking faults represent deviations from a regular layer sequence, turbostratic disorder reduces three-dimensional coherence through random translational or rotational relationships between layers, and interstratification involves the coexistence of different repeat distances or stacking arrangements within one crystallite. These contributions are not equivalent and may affect different reflection families unequally [134,135].

Simulated and experimental XRPD patterns have shown that LDHs may contain intergrowths of 3R_1-_ and 2H_1-_like sequences rather than a single ideal polytype [132]. Stacking faults can modify peak width, position, intensity, and asymmetry, particularly for non-basal reflections, and their effects may be concealed by a single isotropic crystallite size parameter [136,137]. Therefore, the assignment of a highly ordered polytype should require consistency across the diffraction pattern rather than reliance on basal reflections alone.

Scherrer analysis estimates the dimension of coherently diffracting domains along the scattering vector after correction for instrumental broadening, rather than the physical size of a platelet or aggregate. Values derived from basal reflections primarily describe coherence along the stacking direction, whereas in-plane reflections probe coherence within the hydroxide sheets. Several coherently stacked nanosheets may contribute to one diffraction domain, while an individual platelet may contain multiple domains. Diffraction-derived values should therefore be compared with AFM or TEM dimensions and should not be reported generally as particle size [138,139].

An analysis of multiple reflections can provide additional constraints on anisotropic broadening. Williamson–Hall-type approaches may offer an approximate separation of size- and strain-related contributions under suitable assumptions, but their interpretation should be approached with caution for highly anisotropic, faulted, or interstratified LDHs. Anisotropic whole-pattern approaches or explicit simulations of faulted layered structures are more appropriate when stacking disorder is substantial. Refinement using an ideal ordered structural model may reproduce average peak positions while failing to account for asymmetric broadening, diffuse scattering, interstratification, or anisotropic coherence [137].

#### 4.1.2. Basal Periodicity and Interlayer Assignment

Basal spacing depends on interlayer anion identity, orientation, hydration, and exchange state and is therefore not a unique fingerprint of a particular anion. Mixed hydration states, partial exchange, or irregularly alternating interlayers can produce broadened, asymmetric, or split basal reflections. Humidity-controlled, variable-temperature, or in situ diffraction may be required to distinguish hydration changes from phase coexistence [140,141].

Unit cell parameters should be assigned according to the identified polytype. The relation c = 3d_003_ applies only when a three-layer rhombohedral structure and the (003) indexing are justified. For uncertain or strongly faulted stacking, reporting the measured basal repeat, d_basal_, is more appropriate than assigning a precise c parameter. Intense (00l) reflections alone do not demonstrate superior crystallinity because their intensities can be strongly affected by preferred orientation and measurement geometry [142,143].

Basal spacing changes alone are also insufficient to confirm intercalation. XRD should be combined with methods that establish the chemical composition and state of the interlayer, such as FTIR or Raman spectroscopy, thermal analysis, elemental analysis, ion chromatography, and, where appropriate, XPS, solid-state NMR, or synchrotron methods [144,145,146]. Analysis of anions released or consumed during exchange can provide particularly useful complementary evidence. Microscopy can establish particle morphology and, in selected cases, local structural information, but it cannot independently determine the chemical identity or average occupancy of interlayer species.

#### 4.1.3. Phase Composition and Hidden Fe-Rich Phases

Phase purity is particularly difficult to establish in Fe-containing LDHs. The absence of additional sharp peaks cannot exclude poorly crystalline, X-ray-amorphous, low-abundance, or strongly overlapping secondary phases. Practical detection limits vary with impurity concentration, scattering power, crystallinity, peak overlap, and preferred orientation. Ferrihydrite-like material, Fe-rich hydroxides, and poorly ordered mixed-metal oxyhydroxides may therefore remain undetected while contributing substantially to adsorption or catalysis [147,148,149,150].

Identification of such phases requires complementary Fe-sensitive or local-structure methods. ^57^Fe Mössbauer spectroscopy can probe Fe oxidation states and hyperfine environments; Fe K-edge XANES is useful for oxidation-state assessment, and EXAFS for local Fe-O and Fe–metal coordination. Pair distribution function analysis can reveal short-range order in nanocrystalline or amorphous domains. Raman spectroscopy and XPS can provide supporting information on vibrations and surface speciation, while STEM-EDS and carefully controlled STEM-EELS can reveal nanoscale segregation and spatial variations in Fe valence [144,146,148,150,151]. Complementary methods can constrain possible phase compositions, but no single combination guarantees the complete exclusion of poorly crystalline Fe-rich impurities.

The importance of phase evolution is especially evident in structurally or redox-sensitive Fe-containing layered hydroxides. CaFe-LDHs may dissolve and reprecipitate as Ca- or Fe-containing phases, including ferrihydrite-like products, calcite, hydroxyapatite, or contaminant-bearing solids [149]. MnFe-LDHs can release Mn or form MnFe_2_O_4_ and birnessite-associated products depending on oxidation state and interlayer chemistry [58]. Green rust may transform into magnetite during aging, lepidocrocite after oxygen exposure, goethite during some reduction reactions, or contaminant-containing Fe(III) precipitates [152,153].

Pollutant removal may therefore involve a dynamic multiphase system rather than an unchanged, phase-pure LDH. Transformations in other M-Fe systems should be demonstrated by before-and-after or operando characterization and not inferred from Fe content or broad XRD reflections alone.

Crystallinity should not be treated as intrinsically beneficial or unfavorable. Greater structural order may improve phase stability and reproducibility, whereas small coherent domains, thin nanosheets, edge-rich structures, or selected defects may increase site accessibility. However, broad reflections alone do not prove the presence of useful vacancies, ultrathin sheets, or high reactivity; they may instead indicate stacking disorder, mixed phases, partial amorphization, or instability. Functional interpretation therefore requires correlation of diffraction with microscopy, surface area measurements, chemical analysis, and performance data [25,92].

Crystallinity and phase composition should also be examined after use. Depending on composition, interlayer chemistry, pH, hydration, and redox conditions, M-Fe LDHs may retain their layered framework, exchange interlayer species, reconstruct, dissolve, oxidize, or form oxyhydroxides, oxides, and contaminant-containing precipitates. In catalytic, electrochemical, and redox systems, the as-synthesized LDH may act mainly as a precursor to the active phase. Fresh, reacted, and regenerated materials should therefore be compared by post-reaction characterization and, where feasible, in situ or operando diffraction and spectroscopy [72,119,149].

A reliable structural assessment should therefore establish, as far as the available evidence permits, whether a layered phase has formed, which stacking model is consistent with the diffraction data, what contributions dominate peak broadening, whether crystalline or poorly ordered secondary phases are present, and whether the initially characterized structure persists under relevant conditions. These questions should be resolved through complementary diffraction, compositional, spectroscopic, and microscopic evidence rather than through any single structural descriptor.

### 4.2. Nanosheet Thickness, Particle Size, and Aggregation

Well-developed M-Fe LDH phases commonly consist of plate-like primary crystallites, including nanoflakes, nanoplatelets, and single- or few-layer nanosheets. However, poorly ordered, transformed, calcined–reconstructed, or multiphase materials may not retain a clearly resolved platelet morphology. Primary units may remain discrete, stack face-to-face, aggregate irregularly, or assemble into hierarchical secondary structures. Primary platelet dimensions should therefore be distinguished from secondary aggregate size: a micrometer-scale aggregate may remain nanostructured when it consists of nanoscale platelets, whereas the dimensions of the aggregate do not directly describe the dimensions of its constituent layered domains [79,154,155].

Nanosheet thickness determines the geometric fraction of metal centers located near external surfaces and influences transport distances through individual layered domains. Thinner or exfoliated nanosheets can increase the external surface-to-volume ratio and reduce diffusion distances, but these geometric advantages do not necessarily translate into greater reactive-site accessibility. Lateral size, aspect ratio, porosity, defect population, aggregation, interlayer chemistry, hydration, and electronic structure may change simultaneously during thinning or exfoliation [92,156,157,158]. Ultrathin nanosheets may also undergo restacking, aggregation, dissolution, or structural transformation more readily than thicker platelets. Nanosheet thickness should therefore be treated as a structural descriptor rather than as a direct measure of accessible or operational reactive sites.

Nanosheet thickness is most directly estimated from AFM step-height profiles of isolated sheets deposited on flat substrates. However, these values should be reported as apparent step heights because adsorbed water, residual ions or organic species, substrate roughness, nanosheet deformation, and tip-sample interactions can affect the measured height. TEM and HRTEM provide complementary information on sheet morphology, lateral dimensions, stacking, lattice fringes, defects, and local pores, but routine top-view TEM images do not provide unambiguous thickness values. Estimates from folded edges or edge-on platelets are useful only when the orientation and the number of stacked layers can be resolved reliably [79,159,160]. Diffraction-derived stacking coherence, AFM step height, and TEM-observed platelet dimensions therefore describe related but non-equivalent structural quantities and should not be used interchangeably.

Lateral platelet dimensions and secondary particle size likewise require separate interpretation. Decreasing lateral platelet size increases the geometric edge-to-basal surface ratio and can shorten transport distances within individual particles. It does not, however, establish a proportional increase in accessible edge environments because nanoscale platelets may form dense aggregates or restacked structures that bury surfaces and restrict interparticle transport. Conversely, larger secondary particles can retain a substantial accessible surface area when assembled from loosely connected platelets with open pore networks. Particle size effects should therefore be interpreted together with aggregation state, packing architecture, porosity, and dispersion rather than through size alone [24,156,161].

Aggregation is a central morphological issue for M-Fe LDHs. Primary platelets and exfoliated nanosheets can undergo face-to-face restacking or form more irregular agglomerates because of charge screening by dissolved ions, counterion-mediated interactions, hydrogen bonding, van der Waals attraction, and capillary forces generated during drying. Depending on the conditions, this process may produce compact lamellar stacks, disordered clusters, or more open edge-to-face and loosely packed three-dimensional platelet networks.

The structural consequence of aggregation depends on aggregate architecture. Dense aggregation, in contrast, decreases the accessibility of basal surfaces and edge sites, blocks interlayer entrances, and increases diffusion resistance. The functional effect therefore depends not only on aggregate size but also on packing density, platelet orientation, pore connectivity, and the accessibility of interparticle voids. In electrochemical model systems for MnFe-LDHs, aggregated nanoparticles and excessively large leaf-like structures have been associated with restricted access to electroactive sites, whereas more uniformly organized nanosheets have provided more effective ion transport. Open networks of interconnected CoFe-LDH nanosheets can similarly retain accessible surfaces and short transport pathways. Apparently, similar secondary morphologies may therefore enhance or reduce performance depending on whether they form open hierarchical porosity or inaccessible compact clusters [162,163,164]. Such performance differences should nevertheless be interpreted together with composition and structural state rather than attributed to aggregate morphology alone.

Morphology is commonly evaluated using complementary electron and probe microscopy techniques. SEM is useful for examining secondary particle shape, aggregate size, surface texture, and supported structures. TEM provides higher-resolution information on individual platelets, overlapping or stacked sheets, folded and edge-on regions, and local structural heterogeneity. HRTEM and electron diffraction can reveal lattice fringes, crystallinity, defects, and crystallographic orientation, whereas elemental distributions should be evaluated specifically by SEM-EDS, STEM-EDS, or STEM-EELS.

An important limitation is that most conventional microscopy characterizes a small, dried, substrate-supported fraction of the material rather than its native hydrated or operating state. Specimen deposition, washing, solvent exchange, and drying can alter platelet spacing, induce restacking, collapse weakly connected porous structures, or change the apparent aggregation state. Consequently, morphology observed by SEM, conventional TEM, or ex situ AFM should not automatically be assumed to represent particle organization in the original suspension. Where aggregation under operating conditions is important, microscopy can be complemented by suspension-sensitive approaches such as dynamic or static light scattering, scattering methods, sedimentation measurements, or appropriately designed cryogenic techniques, with the choice depending on particle size, concentration, and dispersion state.

Electron beam effects introduce an additional limitation for redox-sensitive Fe-containing phases. In sulfate green rust, for example, low-dose TEM imaging largely preserved the plate-like morphology, whereas increasing electron fluence caused substantial oxidation of structural Fe(II) [79,151,160]. Cryogenic or anoxic specimen preparation and low-dose acquisition should therefore be considered for sensitive Fe(II)-containing materials, and beam-induced transformations should be distinguished from intrinsic structural heterogeneity.

Taken together, nanosheet thickness, lateral platelet size, aggregate size, and aggregation architecture represent distinct levels of M-Fe LDH morphology and should not be collapsed into a single concept of “particle size”. Their relevance to reactive-site accessibility depends on whether geometrically available surfaces remain exposed in the hydrated or operating state of the material. A rigorous morphology–accessibility relationship therefore requires distinction among primary platelet dimensions, secondary aggregation, dry-state porosity, suspension state organization, and the structural state present during operation.

### 4.3. Porosity, Surface Area, and Accessibility of Reactive Sites

Porosity and surface area influence contact between M-Fe LDHs and pollutants, oxidants, electrolyte ions, and reaction intermediates. However, nitrogen physisorption characterizes the texture accessible to N_2_ at 77 K in a dried and degassed material. It does not directly describe hydrated interlayers, aqueous aggregate structure, or the population of chemically active sites under operating conditions. Gas sorption data should therefore be interpreted together with morphology, interlayer chemistry, hydration, aggregation, and application-specific accessibility [124,160,165].

#### 4.3.1. Gas Physisorption Methodology

Sample pretreatment is a major source of uncertainty because degassing is not necessarily structurally neutral for LDHs. The removal of surface and interlayer water may alter basal spacing, hydrogen-bonding networks, and interlayer anion orientation. Excessive heating or vacuum treatment may initiate dehydroxylation, anion decomposition, or structural collapse, whereas insufficient degassing may leave residual adsorbates and decrease the apparent nitrogen-accessible area. Degassing temperature, duration, pressure, and heating program should therefore be reported and selected using thermal or variable-temperature structural data for the specific material [145,165,166].

The BET fitting interval should not be selected automatically. In particular, the conventional range of $\left(\frac{P}{P_{0}}=0.05-0.30\right)$ is not universally valid. The selected region should satisfy the Rouquerol consistency criteria, $n\left(1-\frac{P}{P_{0}}\right)$, should increase monotonically, the BET constant, C, should be positive, and the pressure corresponding to the calculated monolayer capacity should lie within the fitted interval. This pressure should also be reasonably consistent with the value calculated from C and with the isotherm knee. A high regression coefficient alone does not establish a physically meaningful BET fit [165]. Reports should specify the adsorptive, measurement temperature, and pretreatment conditions, in addition to the fitting range, the number of points, the BET constant, and preferably the complete adsorption–desorption isotherm [165,167,168].

Pore volume and pore size distributions are model-dependent. The BJH method relies on simplified assumptions regarding capillary condensation and pore geometry and may misrepresent narrow mesopores, irregular networks, and slit-like voids between nanosheets. Adsorption and desorption branch results may differ because of pore blocking, cavitation, and network effects [165,169,170]. DFT-based methods may improve nanopore analysis, but only when the selected kernel is appropriate for the adsorptive, temperature, surface chemistry, and pore geometry [171]. Pore size distributions should therefore be interpreted as model-dependent structural estimates rather than direct images of a unique pore network.

Hysteresis loop classification should likewise remain qualitative. H3-type hysteresis is consistent with non-rigid aggregates of plate-like particles and slit-like interparticle spaces, but it does not uniquely demonstrate uniform slit-shaped mesopores. Similar loop shapes may also result from incompletely filled macroporous networks, pore blocking, cavitation, or collective filling and emptying. Specific pore geometries should therefore be supported by microscopy, scattering, or suitable pore network analysis [165,172]. Therefore, specific pore architectures should be supported by complementary morphological or scattering evidence rather than inferred from hysteresis shape alone.

#### 4.3.2. Dry-State Porosity and Aqueous Accessibility

A substantial fraction of the mesoporosity measured in dried LDH powders may originate from voids between aggregated or restacked platelets rather than permanent pores within individual crystallites [173]. Solvent exchange, interlayer chemistry, suspension concentration, redispersion conditions, and drying route can strongly affect restacking, exfoliation, and the resulting textural properties. Freeze-, spray-, and oven-dried materials may therefore exhibit different BET areas and morphologies even when their layered structures remain detectable by XRD [118,174]. BET area should therefore be regarded partly as a property of the preparation and drying history of the measured powder, rather than solely as an intrinsic property of the LDH structure.

Nitrogen accessibility at 77 K should not be equated with accessibility to water, hydrated ions, or dissolved organic molecules. Hydration, swelling, ion exchange, and reconstruction may create aqueous pathways that are absent in the dry material. Conversely, narrow or strongly hydrated galleries may participate in ion exchange while remaining inaccessible to nitrogen [135,175]. Interlayer participation should instead be examined using water–vapor sorption, ion exchange measurements, humidity-controlled or in situ XRD, anion mass balance, and characterization of the reacted solid [176]. Adsorption capacity and kinetic fitting alone cannot distinguish between external adsorption, interlayer exchange, secondary precipitation, and bulk reconstruction [177].

The relevance of interparticle porosity also depends on the application. Such voids may facilitate transport in electrodes, packed beds, immobilized composites, or catalyst layers, but may change substantially after redispersion in water. Aqueous aggregation is affected by ionic strength, pH, and specific adsorption of carbonate, sulfate, phosphate, and other ions [178]. Consequently, dry-state gas sorption should not be used alone to infer transport pathways in suspension. Where such behavior is central to the interpretation, methods capable of probing dispersion or particle organization in the hydrated state should provide complementary evidence.

Porosity and accessibility should also be evaluated after use because adsorption, precipitation, pore blocking, aggregation, dissolution, ion exchange, and surface reconstruction can alter the material [148,179]. Post-reaction BET changes must be interpreted cautiously because recovery, washing, redrying, and degassing can themselves modify platelet restacking. Fresh and spent samples should therefore be treated using identical protocols and compared together with XRD, microscopy, spectroscopy, elemental analysis, and dissolved metal measurements. A decrease in BET area may indicate pore blocking, restacking, or particle loss, whereas an increase may result from fragmentation, dissolution–reprecipitation, or formation of a new porous phase. Gas sorption alone cannot distinguish these mechanisms [4,118,137].

#### 4.3.3. Physical Area Versus Chemically Accessible Reactive Sites

A high BET surface area is not equivalent to a high density of chemically accessible or catalytically active sites. Nitrogen-accessible area may include relatively inert basal planes, carbonate-blocked regions, residual synthesis species, or pores inaccessible to hydrated target molecules. Conversely, an LDH with a moderate BET area may contain chemically accessible surface hydroxyl environments, exchangeable interlayers, undercoordinated edge environments, redox-active M-Fe pairs, defects, or reconstructed active phases [51,124]. These environments should nevertheless be regarded as candidate or potential reactive sites until their accessibility and role in the relevant process are independently demonstrated.

Physical and chemical accessibility should therefore be distinguished. A reactive center must be geometrically accessible via an external surface, a layer edge, a hydrated interlayer, a pore, or an open aggregate network. It must also possess the required charge, protonation state, coordination environment, oxidation state, and chemical affinity. Changes in pH, potential, or electrolyte composition may strongly alter surface complexation, anion exchange, or redox activity without producing a corresponding change in BET area [180].

Application-specific methods are needed to estimate operationally accessible sites. Acid-base titration, anion-exchange capacity, probe molecule uptake, surface-hydroxyl analysis, and electrochemical redox measurements may provide useful estimates, although the resulting values depend on the selected probe, electrolyte, pH, potential, and assumed site model. XPS, XANES, EXAFS, and Mössbauer spectroscopy can identify oxidation states, local coordination, and phase distributions, but they do not independently provide a universal active-site density [181,182]. Site accessibility should therefore be treated as an operationally defined property rather than as a single material constant.

The distinction is particularly important when normalizing functional data. In electrocatalysis, the electrochemically active surface area does not necessarily scale with the number of redox-active metal centers, and its value may depend on the catalyst state and the applied potential. In adsorption, uptake may be governed by surface charge, ligand exchange, inner-sphere complexation, interlayer exchange, or precipitation rather than by geometric area. Rates or capacities normalized only to BET area should therefore not be interpreted as intrinsic site-specific activity unless the identity and accessibility of the relevant sites have been independently established [182,183].

Area normalization is most defensible within a closely related series synthesized, pretreated, and measured under identical conditions. Even within such a series, differences in metal distribution, oxidation state, hydroxyl density, defect population, interlayer chemistry, and affinity for the target species should be considered. Mesoporosity is beneficial only when it provides connected, wetted, and stable transport pathways. Its contribution depends on pore connectivity, tortuosity, wetting, aggregate stability, and the hydrated dimensions of the reacting species. Claims that increased BET area or mesoporosity directly improves performance should therefore be supported by kinetic, diffusion, or chemical accessibility evidence [184,185,186].

Conventional characterization methods provide different levels of structural information and should not be treated as interchangeable evidence. Broad PXRD reflections, high BET surface area, nanosheet morphology, or fitted XPS components may support structural interpretation but do not independently establish defect density, reactive-site accessibility, or active-site identity. Stronger assignments require complementary phase-sensitive, wet-state, probe-based, and, where appropriate, in situ or operando characterization (Figure 4).

Arrows indicate increasing evidential strength rather than a reaction sequence. Colors, schematic profiles, and structural symbols are illustrative only and do not represent specific M-Fe systems or samples. The warning panel summarizes observations that are insufficient on their own to establish active-site identity.

### 4.4. Defect Chemistry, Redox Heterogeneity, and Surface Hydroxylation

Structural and chemical heterogeneity in Fe-containing layered hydroxides extends beyond what is captured by descriptors such as crystallinity, surface area, or particle dimensions. Cation vacancies, hydroxyl-related defects, edge undercoordination, local compositional heterogeneity, mixed-valence states, and variations in surface hydroxylation can generate chemically distinct local environments. Stacking disorder may also modify the exposure and accessibility of such environments without necessarily constituting a local point defect. These features may be concentrated at particle edges, exposed basal surfaces, interlayer entrances, reconstructed surface domains, and interfaces with secondary phases. Mixed-valence states may also be intrinsic to the layered structure, as in Fe(II)/Fe(III) green rust [9,11,94]. Their structural presence, however, should be distinguished from evidence of their accessibility or operational activity.

Defect terminology should be used cautiously and should reflect the host phase. Unlike metal oxides, where an oxygen vacancy denotes the absence of lattice O^2−^, the anionic sublattice of brucite-like LDHs consists mainly of OH^−^ groups. Defects in as-synthesized LDHs are therefore more appropriately described as hydroxyl or cation vacancies, deprotonated oxygen species, distorted metal coordination, or undercoordinated edge sites. The term “oxygen vacancy” requires particular caution because an O-deficient environment in a hydroxide cannot automatically be interpreted using the defect chemistry of an oxide lattice. Oxygen-vacancy terminology may become more appropriate after dehydroxylation, calcination, or reconstruction into oxide- or oxyhydroxide-like domains, but the host phase in which the defect is assigned should be identified explicitly [94,187].

Defect assignment should therefore rely on converging, phase-appropriate evidence rather than on a single non-unique spectroscopic feature. Depending on the proposed defect, useful constraints may be provided by EPR, XANES/EXAFS, total-scattering or PDF analysis, Raman spectroscopy, quantitative composition, controlled defect-generation experiments, microscopy, or DFT-supported structural interpretation [94,187]. The appropriate evidence depends on defect type: methods capable of supporting a cation-vacancy assignment are not necessarily sufficient to establish hydroxyl deficiency, local coordination distortion, or an electronically distinct edge environment.

When independently established, defect populations can modify local coordination, surface acid-base behavior, metal–metal interactions, or the distribution of hydroxyl environments. These changes may alter adsorption, electron transfer, oxidant interaction, or structural reconstruction, but the direction and magnitude of the effect depend on defect identity, concentration, spatial distribution, metal composition, oxidation state, and interlayer chemistry [58,94,104]. Increasing disorder should therefore not be treated as a general strategy for increasing reactive-site density. Beyond a system-dependent range, structural disorder may instead promote dissolution, aggregation, metal redistribution, or the formation of secondary oxide and oxyhydroxide phases.

In catalytic and electrochemical systems, cation vacancies, edge undercoordination, and defects within reconstructed oxyhydroxide domains may modify electronic transport and expose reactive metal centers. They may also facilitate hydroxide adsorption and surface reconstruction into catalytically active oxyhydroxide-like phases [72,188,189]. Vacancies should therefore not be considered only as static structural imperfections. Under operating conditions, they may participate in the formation or stabilization of the active surface phase. Nevertheless, evidence obtained for reconstructed or LDH-derived phases should not be attributed automatically to the pristine LDH lattice.

Application studies illustrate this distinction but should be interpreted cautiously. For oxyanion adsorption, accessible edge, surface-hydroxyl, and interlayer sites may participate in ligand exchange, ion exchange, and inner-sphere complexation. Their contribution should not be described as defect-controlled unless vacancies or undercoordinated metal centers have been identified independently and correlated with uptake [190,191]. During persulfate or peroxymonosulfate activation, deliberately introduced cation or oxygen vacancies in NiFe-LDHs have been associated with changes in oxidant adsorption, surface electronic structure, and interfacial electron transfer. In electrochemical systems, defect-rich NiFe-LDHs may undergo faster surface reconstruction and exhibit improved charge-transfer kinetics [27,192]. Enhanced activity should nevertheless be evaluated together with cycling stability, dissolved metal concentrations, and changes in surface composition and phase structure. Operando and post-reaction studies show that Fe dissolution, redistribution, and redeposition can either deactivate NiFe oxyhydroxides or, when suitable cation defects are present, contribute to the recapture and stabilization of active Fe sites [188,193].

Mixed-valence states provide another important source of reactivity. In as-synthesized M(II)-Fe(III) LDHs prepared from Fe(III) salts, Fe should generally be considered predominantly Fe(III) unless spectroscopic evidence indicates partial reduction or the presence of a mixed-valent secondary phase. Under catalytic or electrochemical conditions, however, both Fe and the second metal may undergo redox changes. Co, Mn, Cu, and Ni centers can participate in electron-transfer processes that influence oxidant activation, pollutant transformation, and the formation of reconstructed active phases.

Redox heterogeneity represents a related but distinct source of local chemical diversity. In conventional M(II)-Fe(III) LDHs synthesized from Fe(III) precursors under nonreducing conditions, Fe(III) is the chemically expected dominant Fe state unless direct evidence supports partial reduction, mixed valence, or a mixed-valent secondary phase. Under synthesis, storage, or operating conditions, however, Fe and the second metal may undergo changes in oxidation state, particularly in CoFe, MnFe, CuFe, and NiFe systems. Fe(II)/Fe(III) green rust represents a fundamentally different case because mixed Fe valence is intrinsic to its layered structure rather than necessarily generated during operation [2,10,72].

Redox-state assignments should be supported by methods appropriate to the spatial and chemical scale of the claim. XPS provides valuable surface-sensitive information but should not be used alone to establish bulk oxidation-state distributions or complete metal redox cycles. XANES and Mössbauer spectroscopy can provide stronger bulk-sensitive constraints on Fe oxidation state and local environments, while EPR or other spectroscopies may provide complementary information for selected paramagnetic states. In situ or operando approaches are particularly valuable when oxidation states are expected to evolve during operation. Surface reconstruction, adsorbed intermediates, beam or air exposure, and secondary phases should all be considered as possible contributors to the measured redox response.

Surface hydroxyl groups are important for the activity of Fe-containing LDHs. Depending on solution pH, they may be protonated or deprotonated and can interact with pollutants through electrostatic attraction, hydrogen bonding, ligand exchange, or inner-sphere complexation. Surface hydroxyl groups associated with Fe and the second metal may contribute to the uptake of phosphate, arsenate, chromate, and other oxyanions [9,11,194]. However, direct binding to specific Fe-OH or M-OH sites should be demonstrated experimentally because external surface complexation, precipitation, and interlayer anion exchange may occur simultaneously.

The presence of hydroxyl groups is therefore less informative than their chemical state and accessibility. Hydroxyl-containing sites may remain inaccessible when they are buried within densely restacked aggregates. Strongly bound interlayer carbonate may also restrict anion exchange and reduce access to internal reactive sites [4,145,192]. During catalytic or electrochemical operation, surface hydroxyl groups may additionally participate in oxidant adsorption, proton-coupled electron transfer, or reconstruction into oxyhydroxide-like domains.

No single characterization signal is sufficient to directly link defect chemistry, redox state, or hydroxylation to an operational reactive site. XPS can identify surface metal states and oxygen-containing environments, but O 1s fitting alone does not prove the presence of oxygen vacancies. High-binding-energy components may arise from hydroxyls, deprotonated oxygen species, adsorbed water, carbonate, organic contamination, or different local coordination [195]. Because individual structural claims are often based on non-unique signals, Table 4 summarizes common interpretations in Fe-containing LDHs, the evidence that is insufficient when used alone, and the complementary methods required for more reliable confirmation (Table 4).

The appropriate characterization strategy should therefore be determined by the structural claim rather than by a fixed analytical checklist. PXRD constrains long-range periodicity but cannot independently establish phase purity, defects, or particle dimensions; microscopy resolves morphology but may not represent hydrated aggregation; gas sorption describes dry-state accessible texture but not operational site density; and spectroscopy constrains chemical states without necessarily establishing site accessibility or function. Strong structure–reactivity assignments require convergence among these levels of evidence. Where the material is expected to reconstruct, dissolve, or change oxidation state during operation, characterization of the pristine solid should be complemented by analysis of the reacted state and, where feasible, by in situ or operando measurements.

### 4.5. Structural Reconstruction, Stability, and Phase Transformation During Application

The structure observed in a fresh LDH is not always the structure that operates during application. Binary Fe-containing LDHs may undergo interlayer ion exchange, changes in hydration, surface reconstruction, dissolution, oxidation or reduction, and precipitation of secondary oxides, oxyhydroxides, or pollutant-containing products. These processes may generate an active working phase, contribute directly to pollutant immobilization, or cause progressive loss of material integrity. Their effects therefore depend on the LDH composition, reaction conditions, and intended mode of operation [58,72,149].

Three transformation regimes are useful for interpreting these changes: controlled reconstruction, reactive or sacrificial transformation, and irreversible degradation. They represent operational categories rather than universally discrete mechanisms, and transitions between them may occur as reaction conditions or cycle number change.

A clear distinction should be made between controlled reconstruction, reactive or sacrificial transformation, and irreversible degradation. Controlled reconstruction is a reproducible conversion of the initial material into a defined active phase while the bulk material, supported catalyst layer, or electrode retains sufficient physical integrity. It should involve limited and acceptable metal release, reach a stable state after activation, and either be reversible when operating conditions change or remain stable during subsequent operation. Controlled reconstruction does not demonstrate preservation of the original LDH phase, but it may provide operational durability of the reconstructed material [72,137,196].

Reactive or sacrificial transformation occurs when the consumption or conversion of the initial layered phase directly contributes to the intended function. Dissolution followed by precipitation of a stable contaminant-containing product is one example. Such behavior may be functionally effective, but it should not be described as the preservation of the original structure or as conventional reusability. Its evaluation instead requires material and contaminant mass balances, identification of transformation products, measurement of dissolved metals, and assessment of the persistence and environmental stability of the final solids.

Irreversible degradation describes progressive structural or compositional deterioration that is not required for the intended function and compromises continued operation. Relevant indicators include continued dissolution, uncontrolled phase segregation, loss of recoverable material or catalyst loading, substantial changes in the metal ratio, collapse of the accessible structure, or progressive loss or alteration of performance [158,193,197]. Transient increases in activity caused by corrosion, dissolution, fragmentation, or exposure of previously inaccessible material should therefore not be interpreted as improved stability unless a reproducible working state is subsequently established.

These categories clarify why different Fe-containing layered hydroxide families require different definitions of stability. In reversible adsorption or ion-exchange systems, the layered framework may remain largely preserved while interlayer species and surface hydroxyl environments participate in uptake. Reuse then requires the recovery of sufficient accessible structure and restoration of functional capacity after desorption or regeneration. Capacity retention alone is incomplete evidence if repeated cycling is accompanied by progressive dissolution, the loss of recoverable mass, pore blocking, or the irreversible accumulation of reaction products [11,119,186].

In catalytic, electrochemical, and advanced oxidation systems, structural changes may form part of the working mechanism. Surface metal centers may change oxidation state, local coordination may evolve, and the outer layers may reconstruct into oxyhydroxide-like domains [72,192,198]. The initial LDH remains relevant because it controls metal dispersion, composition, nanosheet morphology, and the accessibility of the reconstructed region. Nevertheless, the active phase under operating conditions should not be assumed to be identical to the as-synthesized LDH.

NiFe-LDH is a representative example of dynamic reconstruction. Under anodic conditions, the initial layered material may convert into a hydrated NiFe oxyhydroxide-like active phase, accompanied by changes in oxidation state, local coordination, interlayer composition, and hydration [72,188,199]. Such conversion may be classified as controlled reconstruction only when the active phase forms reproducibly, the electrode or catalyst layer remains intact, and metal loss becomes limited after activation. Recent studies also show that Fe may dissolve, migrate, redeposit, or segregate during operation. Therefore, apparent activity retention does not necessarily demonstrate compositional stability, and reversible Fe dynamics should be distinguished from progressive Fe depletion or formation of inactive Fe-rich phases [158,192,196,197].

CaFe hydrocalumite-/AFm-related layered hydroxides illustrate a different regime. Their environmental function may involve dissolution of the initial layered phase followed by precipitation of Ca- or Fe-containing products. Phosphate removal, for example, may involve the formation of new calcium phosphate phases rather than adsorption only on an intact CaFe-LDH surface [5,149,200]. This process is better described as a reactive phase transformation. Evaluation should include dissolved Ca and Fe, solution pH, phosphate mass balance, recovered solid mass, and direct characterization of the newly formed products. High pollutant removal cannot, by itself, demonstrate the preservation or reusability of the original CaFe material.

Green rust is another system in which transformation is intrinsic to its function. Its structural Fe(II) acts as a reducing component, and pollutant reduction or exposure to dissolved oxygen may convert the layered phase into magnetite, ferrihydrite, feroxyhyte, goethite, lepidocrocite, or mixed Fe-containing products [201,202,203]. Green rust may therefore function as a sacrificial reductant rather than a structurally stable reusable adsorbent. The extent of Fe(II) consumption, Fe dissolution, pollutant speciation, final mineralogy, and stability of the pollutant-containing products should be determined. Because green rust is highly sensitive to oxidation, anoxic handling and time-resolved or in situ characterization are particularly important.

Calcination–rehydration provides another example in which recovery of a layered diffraction pattern does not necessarily represent restoration of the original material. Calcination converts LDHs into mixed-metal oxide phases, while rehydration in anion-containing solutions may partially restore layered domains through the memory effect. However, reconstruction does not necessarily reproduce the initial LDH because interlayer composition, metal distribution, crystallinity, porosity, surface chemistry, and secondary-phase content may change [145,204,205]. These materials should therefore be described as reconstructed LDH-derived phases rather than unchanged or fully recovered original LDHs.

Stability should be treated as a quantitative, application-specific property. At minimum, reuse or durability studies should report the percentage of performance retained under identical operating conditions, dissolved Fe and second-metal concentrations, recovered solid mass or retained catalyst loading, and changes in bulk and surface metal ratios. Where relevant, they should also quantify retained crystalline phase fractions, changes in morphology, BET surface area and pore volume, accumulation of pollutants or reaction products, and reversibility after regeneration. For materials containing Co, Cu, Ni, Mn, or other potentially hazardous metals, the toxicity of the leachate and transformed solids should also be evaluated. In electrocatalytic systems, current or potential drift should be combined with quantitative metal-dissolution measurements rather than being used as the sole durability criterion [206].

Retention of several basal reflections after application is not sufficient evidence of structural stability. It only demonstrates that some ordered layered domains remain within the recovered bulk sample. Conventional ex situ XRD may not detect surface-localized reconstruction, amorphous products, minor secondary phases, changes in local coordination, or selective loss of one metal. Retained basal peaks also do not demonstrate the preservation of accessible surface sites, original porosity, or initial metal stoichiometry. XRD should therefore be combined with solution analysis by ICP-OES or ICP-MS, bulk and surface elemental analysis, XAS or Mössbauer spectroscopy where appropriate, microscopy, Raman or FTIR spectroscopy, and mass recovery. Quantitative phase analysis with an internal standard, total-scattering methods, or pair distribution function analysis may provide stronger evidence when partial amorphization or local reconstruction is expected [137,193].

A useful state-based framework therefore distinguishes the as-synthesized state, the active or working state, and the recovered state, with a regenerated state evaluated separately when reuse is claimed. These states may remain similar in reversible adsorption systems but differ substantially in reconstructing catalysts, CaFe reactive materials, and green rust. Comparison among them should establish whether the observed transformation is controlled and operationally durable, functionally sacrificial, or progressively degradative. This distinction is necessary for identifying the actual active phase, evaluating reuse potential, and determining whether pollutant removal transfers contaminants into a stable solid or creates additional dissolved and solid waste (Figure 5).

The as-synthesized material may evolve into an active/working state and, after operation, follow controlled reconstruction, reactive/sacrificial transformation, or irreversible degradation pathways. A regenerated state should be treated as a distinct material state because recovery of function does not necessarily imply the restoration of the original structure or composition. The three pathways classify the structural evolution identified during and after the operation rather than representing transformations initiated only after material recovery.

Roman numerals I–IV indicate successive material states. Arrows show possible directions of material evolution during operation, recovery, regeneration, transformation, or degradation. Colors are used only to distinguish the different material states and transformation pathways and do not represent specific M-Fe systems.

## 5. Research Gaps and Future Priorities in Synthesis–Structure Relationships

### 5.1. Standardization and Reproducibility of Coprecipitation Protocols

Coprecipitation is often described as a simple and controllable route for preparing Fe-containing binary LDHs, but it is not a single standardized method. Published protocols differ in precursor and base concentrations, reagent-addition mode, pH control, reactor geometry, mixing, temperature, aging, washing, drying, and storage [99,108]. Consequently, materials assigned to the same nominal M-Fe system can differ in measured composition, interlayer chemistry, structural coherence, aggregation state, and secondary-phase content. Reproducibility therefore requires the control and reporting of the complete synthesis history rather than the replication of nominal precursor ratios and final pH alone.

This sensitivity arises because Fe-containing LDH formation depends on the full precipitation history rather than only the nominal recipe. Syntheses conducted at the same target or final pH may experience different local OH^−^ exposure, supersaturation, carbonate availability, mixing conditions, and reagent-addition histories. These differences are particularly important for Fe(III), which may hydrolyze first to form transient Fe-rich oxyhydroxides before dissolving, recombining with the divalent metal, or persisting as secondary phases [79,99]. Reporting only the final pH therefore does not establish whether the metals precipitated concurrently or how the layered phase developed. The pH trajectory, including deviations during reagent addition and changes during aging, should be reported together with the pH-control and reagent-dosing methods.

Reagent-addition protocols must also be distinguished. Constant-pH co-titration, variable-pH precipitation, rapid base addition, and direct precipitation generate different histories of supersaturation and metal hydrolysis. Direct head-to-head comparisons remain limited, particularly for binary M-Fe LDHs. For ZnFe-LDH, Mendil and Nasrallah reported that constant-pH synthesis produced adsorption capacities 1.09 and 1.31 times higher than direct and variable-pH routes, respectively, along with faster dye removal at a lower dosage [86]. Earlier comparisons of MgAl-LDHs and ZnCr-LDHs similarly found higher crystallinity, smaller particles, larger specific surface area, and greater average pore diameter under constant-pH conditions [207]. These studies demonstrate that the addition mode can alter the resulting material, but they do not establish a universally superior protocol or, on their own, demonstrate batch-to-batch reproducibility. Systematic comparisons should instead determine which aspects of precipitation history control phase identity, measured composition, and nanostructure within each M-Fe system.

Hydrodynamic conditions are another major source of variation and cannot be described by stirring rate alone. The same rpm may generate different local mixing environments depending on vessel dimensions, liquid volume, impeller geometry, baffles, feed location, and fluid properties. In MgAl-LDH synthesis, agitation speed, mixing time, and baffle configuration affected yield, crystallinity, coherent-domain size, surface area, and pore structure, while baffles improved alkaline-feed dispersion and reduced stagnant regions and solid-body rotation [108]. Continuous-flow studies likewise show that particle size, aspect ratio, and crystallinity depend on reactor geometry, shear rate, flow rate, and the relationship between micromixing, nucleation, and growth [208,209]. Therefore, scale-up should preserve relevant mixing and precipitation conditions rather than simply reproduce laboratory rpm. Future studies should increasingly report transferable hydrodynamic descriptors, such as impeller geometry, characteristic mixing time, power input, and other reactor-appropriate parameters, where available.

Nominal precursor composition must be distinguished from the composition of the recovered solid. Selective hydrolysis, incomplete precipitation, oxidation, dissolution during washing, and secondary-phase formation may all alter the final M/Fe ratio. In one comparative MFe-LDH series, feeds containing 0.5 mmol Cu or Mn and 0.125 mmol Fe had a nominal M/Fe ratio of 4, whereas ICP-OES gave Cu_0_._78_Fe_0_._22_ and Mn_0_._78_Fe_0_._22_, corresponding to ratios of approximately 3.55 [210]. Ca-Al-Fe hydrocalumite also showed differences between starting and ICP-measured compositions, while excess Ca in binary CaFe synthesis formed Ca(OH)_2_ rather than proportionally increasing Ca incorporation into the layered phase [42]. Carbonate green rust formed with an Fe(II)/Fe(III) ratio close to 2 regardless of the initial solution ratio, demonstrating even stronger compositional regulation by the solid phase [211]. Both nominal and measured bulk compositions should therefore be reported. Where the oxidation state defines the material, as in green rust, oxidation-state-resolved analysis is additionally required. Agreement of the bulk elemental ratio with the nominal stoichiometry should not, however, be interpreted by itself as evidence that both metals occupy the same layered phase.

Interlayer composition requires similar control. Carbonate may be introduced intentionally through the alkaline reagent or unintentionally from dissolved and atmospheric CO_2_. Even when nitrate or chloride precursors are used, carbonate incorporation can alter basal spacing, hydration, stability, and anion-exchange behavior [2,20]. Reports should specify whether synthesis and handling were performed under air, inert gas, anoxic, or CO_2_-controlled conditions, whether carbonate was intentionally added, and how the interlayer anion was verified.

Washing, drying, and storage should be regarded as part of the synthesis protocol rather than as neutral processing steps. Washing can remove or exchange interlayer ions, alter pH, and dissolve unstable phases, while drying can change hydration, basal spacing, aggregation, texture, oxidation state, and phase composition. Exposure of wet non-carbonate LDHs to air may promote carbonate incorporation, and drying temperature and duration can modify crystallinity and interlayer structure [212]. Green rust is particularly vulnerable to oxidation artifacts during washing, drying, and storage, making anoxic handling and wet-state or in situ characterization preferable where possible [39]. MnFe systems may also transform after synthesis: Mn(II)-containing chloride phases underwent rapid Mn dissolution and MnFe_2_O_4_ formation in aerated neutral suspensions, whereas carbonate exchange or oxidation to Mn(IV) increased their stability [58]. Reproducibility, therefore, requires documentation of the complete pathway from precipitation to the state in which structural characterization is performed.

Based on these limitations, Table 5 proposes a two-level reporting framework. Minimum information is required to reconstruct the chemical and processing history of a synthesis, whereas advanced descriptors are intended for mechanistic studies, scale-up, and interlaboratory validation.

The purpose of standardization is not to impose one universal synthesis route. Different protocols are required to tune crystallinity, morphology, interlayer chemistry, and phase stability. Standardization should instead establish a common level of reporting and validation that allows the precipitation history to be reconstructed and results to be compared across studies. Reproducibility should be demonstrated through independently prepared batches and quantitative reporting of variability in the structural and compositional outputs relevant to the claim being tested. Interlaboratory studies using shared reference protocols and common characterization criteria represent a particularly important future step toward distinguishing genuinely transferable synthesis–structure relationships from laboratory-specific outcomes.

### 5.2. System-Specific Compositional Windows, Terminology, and Phase-Purity Criteria

A major limitation in the literature on binary Fe-containing layered hydroxides is the use of the conventional trivalent-cation fraction (x = 0.20–0.33) as a universal stability window [213]. Although this range is a useful starting point for brucite-like M(II)-Fe(III) LDHs, actual phase boundaries depend on the metal pair, hydrolysis and redox chemistry, interlayer anion, pH, and precipitation pathway [46]. System-specific windows must therefore be established experimentally rather than inferred from a generalized LDH formula.

Such windows require controlled compositional series in which the M/Fe ratio is varied, while other major synthesis variables, including pH trajectory, total metal and base concentrations, reagent-addition mode, interlayer chemistry, atmosphere, temperature, and aging, are held constant or independently quantified. Nominal and measured compositions must also be distinguished because selective precipitation, incomplete incorporation, washing-induced dissolution, oxidation-state changes, and poorly crystalline secondary phases can shift the composition of the recovered material. Importantly, agreement between the nominal and measured bulk M/Fe ratios does not, by itself, establish that both metals occupy the same layered phase. Without phase-sensitive compositional evidence, changes attributed to the M/Fe ratio may instead reflect changes in phase assemblage, local cation distribution, or secondary-phase content.

The need for such studies is particularly evident in CuFe and MnFe systems. A MnFe series spanning nominal Mn/Fe ratios from 1:4 to 8:1, the highest catalytic activity occurred near Mn/Fe = 1:1–2:1 [10]. This observation identifies an application-specific optimum but does not define a single-phase compositional window because morphology, surface area, Mn oxidation state, local cation distribution, and operational phase may vary concurrently. Similarly, selected Cu-containing LDH studies indicate that nominally Cu-rich formulations may produce copper hydroxysalts or Cu(OH)_2_ together with poorly crystalline Fe-rich material [62]. Existing observations should therefore be used to identify regions requiring controlled phase mapping rather than to be converted into universal CuFe or MnFe stability limits.

ZnFe studies further illustrate that LDH-like diffraction does not establish phase purity. Ahmed et al. observed basal LDH reflections across nominal Zn/Fe ratios of 1–4 at pH 8, although ZnO and an additional Fe-containing phase were reported within the series [214]. Because this study also examined calcined ZnO/ZnFe_2_O_4_ products, the precursor-phase assignments should be interpreted only within the reported characterization framework. Qu et al. obtained measured Zn/Fe ratios of 6.47, 1.71, and 0.20; the intermediate composition formed the cleanest Zn-Fe layered phase, whereas Zn-rich and Fe-rich conditions promoted simonkolleite/ZnO and amorphous iron oxide, respectively [215]. Because these studies used different synthesis chemistries, including routes not directly equivalent to conventional aqueous coprecipitation, their apparent boundaries should not be combined into a universal ZnFe phase diagram. A controlled coprecipitation series with measured compositions and quantitative or complementary phase analysis remains necessary.

Structural family distinctions must also be retained. Conventional M(II)-Fe(III) LDHs, CaFe hydrocalumite-like phases, and Fe(II)-Fe(III) green rust should not be evaluated using one crystal chemical model because their layer structures, cation coordination, redox behavior, and transformation pathways differ [80,213,216]. A compositional range meaningful for conventional M(II)-Fe(III) LDHs therefore cannot be transferred directly to CaFe layered hydroxides or green rust.

CaFe hydrocalumite-/AFm-related layered hydroxide materials demonstrate why precursor ratio and layer stoichiometry must be separated. The ordered chloride-intercalated phase has the composition Ca_2_Fe(OH)_6_Cl·2H_2_O and a structural Ca/Fe ratio of 2. In the coprecipitation series reported by Sipiczki et al., the cleanest CaFe layered phase was obtained at a nominal Ca/Fe ratio of 2:1, whereas higher ratios increased Ca(OH)_2_ formation; limited Ca(OH)_2_ was also associated with greater persistence during aging under those specific conditions [40]. In a different Ca(OH)_2_-hydrolysis route, an initial Ca/Fe ratio near 3 promoted precursor conversion and maintained suitable solution chemistry without implying formation of a Ca_3_Fe layer [116]. CaFe phase-purity criteria should therefore distinguish fixed-layer stoichiometry from route-dependent precursor excess and should quantify Ca(OH)_2_ and Fe-rich by-products [216].

A second major gap is the absence of consistent phase-purity criteria. Broad PXRD reflections may arise from small coherent domains, stacking disorder, microstrain, low crystallinity, amorphous material, or multiphase products and should not be interpreted automatically as evidence of vacancies or high defect density. Likewise, the absence of sharp impurity peaks does not exclude ferrihydrite, Mn/Fe oxyhydroxides, copper hydroxides or oxides, Ca(OH)_2_, or oxidized green rust products.

Evidence from Fe-substituted ternary MgAl-LDHs and ZnAl-LDHs demonstrates this methodological risk: Rietveld refinement could not fully reconcile bulk and crystalline compositions, whereas PDF analysis revealed amorphous two-line ferrihydrite [150]. This is not direct evidence for binary MgFe or ZnFe phase behavior, but it shows why conventional PXRD alone may miss poorly crystalline Fe-rich phases. Binary CuFe- and MnFe-containing studies provide complementary examples in which LDH-like reflections coexisted with segregated or transformed products [58,62].

Phase-purity assessment should therefore be claim-driven rather than based on a fixed analytical checklist. PXRD should establish the detectable long-range phase assemblage, while measured bulk composition constrains elemental stoichiometry. Additional methods should be selected according to the most plausible unresolved alternatives. Solution metal balance can reveal incomplete incorporation or selective loss during washing. Raman and FTIR spectroscopy can support identification of interlayer anions, carbonate contamination, and some secondary phases, while microscopy and elemental mapping can detect segregation, although homogeneous EDS maps do not prove atomic-scale cation mixing. Mössbauer spectroscopy and Fe K-edge XANES can assess Fe oxidation states, EXAFS can examine local coordination, and total-scattering or PDF methods can detect short-range order in poorly crystalline phases [38]. The required evidence should therefore depend on the phase-purity claim and on the competing phases that must be excluded.

Terminology should be equally phase-specific. Nominal precursor ratios should not be reported as product compositions without analytical confirmation. Mixed valence is not itself a structural defect, broad PXRD peaks do not prove vacancy formation, and reconstructed oxide- or oxyhydroxide-like phases should not be described as unchanged LDHs. Vacancy terminology should reflect the host phase, and O 1s fitting alone should not be accepted as evidence of oxygen vacancies because high-binding-energy components may arise from hydroxyls, adsorbed water, carbonate, organic contamination, or undercoordinated oxygen-containing species [195,217]. Such assignments require complementary phase-specific evidence.

Future work should, therefore, move from generalized composition rules toward system-specific synthesis–composition–phase maps. For each structural family, these maps should connect nominal precursor composition and synthesis conditions with measured solid composition, phase assemblage, oxidation state, interlayer chemistry, and uncertainty in phase assignment. Negative results and multiphase regions are as important as successful syntheses because they define the boundaries within which meaningful structure–property comparisons can be made. Ultimately, a compositional window should represent a reproducible region of experimentally demonstrated phase behavior rather than a range inferred from nominal stoichiometry or an LDH-like diffraction pattern.

### 5.3. Time-Resolved Formation Pathways and Intermediate Phases

A major research priority for binary Fe-containing LDHs is the direct investigation of their formation pathways. Most studies characterize only the final precipitate, although similar endpoint compositions and PXRD patterns may result from direct co-nucleation, sequential precipitation, precursor-mediated growth, dissolution–reprecipitation, or aging-induced reorganization [37]. Final-product characterization can establish what material was recovered but cannot demonstrate how it formed.

This limitation is particularly important for Fe-containing systems because rapid Fe(III) hydrolysis may generate Fe-rich hydroxides or ferrihydrite-like intermediates before the layered phase develops [79,149]. Depending on pH, local supersaturation, carbonate availability, complexation, and mixing, the second metal may precipitate concurrently, adsorb onto Fe-rich precursors, or incorporate during subsequent reorganization. These pathways can lead to different local cation distributions, interlayer compositions, structural coherence, and secondary-phase assemblages even when the nominal M/Fe ratio and endpoint diffraction pattern are similar. The Fe-rich precursor formation should, nevertheless, be treated as a documented system-specific pathway rather than as a universal mechanism of M-Fe LDH synthesis.

Mechanistic evidence is strongest for only a few systems. CoFe-LDH studies suggest initial formation of nanodispersed ferrihydrite-like species followed by Co adsorption and reorganization into an ordered carbonate-containing phase [37]. This provides a useful example of precursor-mediated formation but should not be generalized to all M-Fe systems. NiFe-LDH studies likewise show that the initial precipitate, aging intermediates, and oxyhydroxide domains generated during electrochemical activation must be distinguished [79,218]. Green rust requires a separate redox-controlled framework because its formation and transformation depend on Fe(II)/Fe(III) ratio, pH, interlayer anion, oxygen exposure, and solution composition [219].

For MgFe-LDHs and ZnFe-LDHs, selected studies suggest that sequential Fe hydrolysis may occur under particular synthesis conditions, but the available evidence is insufficient to establish a general formation pathway. Potentiometric titration of Mg^2+^/Fe^3+^ and Zn^2+^/Fe^3+^ chloride mixtures indicated formation of Fe(III) hydroxide or hydrous oxide before development of a mixed-metal layered phase [220]. In MgFe sulfate-carbonate systems, ferric hydroxide was detected under Fe-rich conditions below pH 9, followed by Mg^2+^ incorporation and LDH crystallization [221]. A ZnFe study in the presence of As(III) proposed sequential formation of ferric arsenate, ferrihydrite, and ZnFe-LDH [222], but arsenic directly modifies Fe precipitation. These observations are therefore condition-specific and cannot define a universal MgFe or ZnFe pathway. Direct in situ measurements combined with solution-phase metal analysis remain necessary.

Time-resolved evidence is similarly limited for MnFe- and CuFe-containing materials. In a Mn(II)-Fe(II)-Fe(III) layered system, the reduced chloride phase released Mn and transformed toward MnFe_2_O_4_ in aerated water, whereas controlled oxidation produced a more stable Mn(IV)Fe(III)-carbonate phase accompanied by birnessite nanorods [57,58]. These ex situ results demonstrate the importance of redox control but do not establish the sequence of events during initial MnFe precipitation. Operando Mn and Fe K-edge XAS, diffraction, solution metal analysis, and redox-potential monitoring are needed to determine when Mn incorporation and oxidation occur.

Selected Cu-containing studies similarly suggest sequential or segregated precipitation under some conditions. pH-resolved studies have reported early Fe-rich hydroxide formation followed by copper hydroxynitrate or Cu(OH)_2_ at higher alkalinity, with Cu-rich crystalline and Fe-rich poorly crystalline products persisting after aging [62,223]. However, much of this evidence derives from multicationic, catalyst-oriented, or ex situ systems. It therefore remains unresolved whether binary CuFe precipitation can proceed via concurrent incorporation under appropriately controlled conditions, whether segregated precursors can reorganize into a homogeneous layered phase, or whether apparent CuFe-LDH formation often reflects persistent nanoscale phase mixtures.

CaFe hydrocalumite-/AFm-related layered hydroxide formation appears to be governed by precursor dissolution and solution speciation rather than the simple coprecipitation of Ca^2+^ and Fe^3+^. For a Ca(OH)_2_-hydrolysis route, modeling supported by HR-XRD and PDF analysis indicated that an initial Ca/Fe ratio near 3 maintained the alkalinity required for Ca_2_Fe(OH)_6_Cl·2H_2_O formation, promoted precursor conversion, and suppressed less-soluble impurities [84,116]. Nucleation was proposed to occur partly on dissolving portlandite surfaces. Conventional coprecipitation studies further showed that excessive alkalinity promoted Ca(OH)_2_ and Fe(OH)_3_ impurities, while phase-pure Ca_2_Fe-LDH could decompose during aging [40]. Direct measurements of Ca and Fe depletion and intermediate-phase composition are still needed to distinguish surface-mediated growth from dissolution–reprecipitation and direct precipitation.

Aging should, therefore, be considered an active stage of phase formation rather than merely a period of crystal growth. It can involve dissolution, reprecipitation, cation redistribution, interlayer exchange, oxidation-state changes, and elimination or persistence of poorly crystalline intermediates. During homogeneous NiFe synthesis, for example, Fe-rich precipitates formed while Ni remained substantially complexed, followed by partial dissolution and progressive appearance of LDH platelets during heating and aging [79,93]. Longer treatment can improve structural coherence during one stage but later promote Ni(OH)_2_, NiO, and NiFe_2_O_4_-containing products under other conditions [109]. Longer aging is therefore not necessarily beneficial, and endpoint characterization may obscure both transient intermediates and, later, over-aging products.

Methodological studies of non-Fe LDHs provide useful experimental frameworks. Simultaneous monitoring of pH, conductivity, light scattering, and synchrotron XRD during MgAl-LDH formation separated initial ion uptake and nucleation from subsequent crystal growth and showed that concentration, temperature, and addition sequence affected induction time and crystallization rate [93]. In-line NIR studies of MgAl-LDHs and ZnAl-LDHs indicated rapid formation of unstable particles followed by slower crystallization and redissolution–reorganization [224], while time-resolved XRD with quenched aliquots identified hydrated hydroxide intermediates during CuCr-LDH formation [225]. These methods are transferable, but the corresponding mechanisms are not, because Fe hydrolysis, metal complexation, and redox chemistry are system-specific.

Future studies should combine complementary time-resolved measurements rather than rely on one technique. Synchrotron XRD can track crystalline-phase evolution, whereas total scattering and PDF analysis can detect poorly crystalline intermediates. XAS can follow metal oxidation state and local coordination, and Raman spectroscopy can identify evolving hydroxide, carbonate, sulfate, oxide, or oxyhydroxide species. Mössbauer spectroscopy is valuable for rapidly quenched Fe-containing intermediates, while solution-phase metal analysis is essential for determining whether metal precipitation is simultaneous or sequential. Redox-potential monitoring and controlled-atmosphere sampling are particularly important for MnFe systems and green rust.

Sampling should cover base addition, pH adjustment, initial precipitation, early and prolonged aging, washing, and storage. Rapid quenching is required because transient Fe-rich or M-rich intermediates may disappear during isolation or aging, while uncontrolled exposure to O_2_ or CO_2_ may generate handling artifacts. The objective is to determine the sequence, lifetime, and transformation of precursor phases and to separate processes occurring during nucleation from those caused by later dissolution, recrystallization, redox change, or interlayer exchange.

Ultimately, formation pathway studies should move from endpoint-based inference toward time-resolved synthesis pathway maps that relate pH trajectory, metal depletion from solution, intermediate-phase identity, oxidation state, and final phase composition. Such maps would establish which mechanisms are reproducible within each M-Fe system and which arise only under particular synthesis conditions, providing the mechanistic basis needed for predictive synthesis rather than empirical recipe optimization.

### 5.4. Quantitative Links Between Synthesis, Nanostructure, and Reactive-Site Accessibility

A central challenge in synthesis–structure studies of Fe-containing binary LDHs is the lack of quantitative links between precipitation conditions, nanostructure, and operationally accessible reactive sites. Functional performance is frequently correlated with broad diffraction features, high BET surface area, thin nanosheets, or hierarchical morphology, although none of these descriptors independently establish defect density, site accessibility, or mass transport. Peak broadening may reflect limited coherent-domain size, stacking disorder, microstrain, or mixed phases, while nitrogen sorption characterizes a dry, degassed solid rather than its hydrated, reactive state [134,165]. Thin or flower-like structures may expose edges and shorten diffusion paths, but they can also restack, aggregate, dissolve, or reorganize in water [148,164]. Quantitative synthesis–structure relationships, therefore, require the separation of structural descriptors from functional interpretation.

Morphological and textural descriptors should therefore be measured independently. Coherent-domain size should be distinguished from particle size, platelet thickness from lateral dimensions, and primary particles from secondary aggregates. External surface area, interparticle porosity, interlayer accessibility, defect population, hydroxyl density, and functional site density should likewise be treated as separate variables rather than interchangeable proxies.

Microscopy needs more quantitative reporting. AFM should provide step-height and lateral-size distributions, the number of objects analyzed, and clear differentiation among primary platelets, exfoliated sheets, and aggregates. SEM and TEM should similarly include particle-size statistics rather than representative images alone. Tyndall et al. analyzed at least 200 NiFe-LDH platelets across at least 20 SEM fields and related lateral size to edge-associated OER activity, although only representative AFM height profiles were reported [156]. Other NiFe-LDH and CoFe-LDH studies reported average or representative nanosheet thicknesses without complete size distributions or explicit sample counts [160,226]. Therefore, future datasets should report not only mean dimensions but also distributions, sample size, dispersion, and, where relevant, covariance between thickness and lateral size.

Gas physisorption data should be treated as supporting textural descriptors rather than direct measures of active-site accessibility. BET analysis should use a physically justified fitting interval, satisfy the Rouquerol consistency criteria, and report degassing conditions, the full isotherm, fitting range, number of points, and BET constant [165]. Pretreatment may alter hydration, interlayer configuration, carbonate content, or hydroxylation, so the relevant methodological and thermal evidence should be cited from the IUPAC guidance and LDH dehydration studies [165,166], rather than from application-specific adsorption comparisons. Pore size models should also match the assumed pore geometry, and interparticle voids should not be equated with permanent pores or hydrated interlayer pathways.

Wet-state characterization should become a routine part of synthesis–structure studies because LDHs may aggregate, disperse, swell, restack, or change surface charge with pH, ionic strength, electrolyte composition, concentration, and aging. DLS, zeta potential or electrophoretic mobility, sedimentation analysis, and, where appropriate, liquid-phase SAXS or cryo-TEM should therefore complement dry-state microscopy. In NiFe-LDH studies, SAXS and electron microscopy identified primary units only a few nanometers in size, whereas DLS and TEM revealed aggregates approaching 100 nm. Size-selected NiFe-LDH dispersions further showed that decreasing platelet size could increase relative edge-site accessibility without a corresponding change in zeta potential [100,156,178]. Such combined measurements are needed to distinguish primary size, reversible aggregation, irreversible restacking, and sedimentation.

Active-site accessibility should be quantified using mechanism-relevant probes rather than inferred from BET area or structural disorder. Depending on the intended function, useful approaches may include acid-base titration, molecular or ionic probe uptake, anion-exchange measurements, redox titration, electrochemical measurements, probe reactions, and spectroscopic monitoring of oxidation-state changes. These measurements remain operationally defined and depend on pH, electrolyte, potential, probe size, and the assumed site model, but they provide a stronger basis for linking synthesis to functionality than geometric area alone.

Independent quantification of hydroxyl accessibility remains particularly limited. Surface hydroxyl abundance is commonly inferred from broad FTIR bands or fitted O 1s components, although these approaches do not directly provide absolute densities of reactant-accessible sites [227,228]. Future studies should distinguish total structural hydroxyl content from proton-exchangeable external groups and from hydroxyl environments accessible to a specific reactant or probe. Combining titration, stoichiometric probe reactions or surface derivatization, and quantitative spectroscopy may provide stronger constraints. Where site-density estimates are reported, they should be expressed per unit mass and, where meaningful, relative to independently determined accessible area, with the assumptions of the site model stated explicitly.

A few NiFe-LDH studies have deliberately introduced metal or oxygen deficiencies through molecular etching, acidic growth control, or reductive treatment and verified coordination loss using XANES and EXAFS rather than XPS alone [104,229]. However, defect generation often changes thickness, composition, surface area, amorphization, or reconstruction simultaneously. Future work should therefore prepare compositionally and morphologically matched defect series, quantify local coordination using complementary phase-specific methods, and correlate defect populations with accessible-site densities and site-normalized rates. Vacancy terminology should remain phase-specific, and O 1s fitting alone should not be accepted as evidence of oxygen vacancies. Mixed valence and reaction-induced oxide or oxyhydroxide phases should likewise be distinguished from defects in the as-synthesized LDH [2,72,230].

The long-term objective should be to move from qualitative correlations toward multivariable synthesis–structure–accessibility datasets. Rather than relating performance to a single descriptor such as BET area or nanosheet thickness, future studies should quantify how synthesis variables jointly influence measured composition, coherent-domain dimensions, platelet morphology, aggregation, hydrated accessibility, local coordination, and operational site density. Statistical experimental design and multivariate analysis can then distinguish correlated descriptors and identify which variables independently predict structural or functional outcomes. Such datasets would provide a stronger basis for predictive synthesis than one-variable-at-a-time correlations.

Support effects should be separated from changes in LDH composition and morphology. Composite preparation may simultaneously alter LDH loading, particle size, aggregation, crystallinity, and exposed area, making it difficult to assign performance changes to the support itself. Studies should therefore compare the unsupported LDH, support, composite, and, where appropriate, a composition-matched physical mixture under identical synthesis and testing conditions. Comparisons should be normalized to LDH or metal content and supported by evidence that distinguishes genuine support-induced changes from altered LDH loading or nanostructure. Detailed application-specific mechanisms involving conductivity, adsorption enrichment, binders, or interfacial charge transfer are better addressed in the application-focused part of the review.

### 5.5. From Empirical Optimization to Predictive Synthesis–Structure Maps

Most studies of binary Fe-containing LDHs still rely on empirical one-variable-at-a-time optimization. Although this approach can reveal basic trends, it is insufficient for predictive synthesis because precipitation variables are strongly coupled. Total metal concentration affects supersaturation; addition rate interacts with mixing to determine local pH; temperature influences hydrolysis, nucleation, growth, and aging; atmosphere controls redox chemistry and carbonate uptake; and complexing agents alter metal speciation, precipitation sequence, and pH trajectory. Apparent effects assigned to one parameter may therefore reflect uncontrolled interactions with others.

These interactions are system-specific. Conditions favorable for MgFe- or NiFe-LDH formation may produce secondary phases in CuFe- or MnFe-containing systems. Carbonate may improve structural stability while reducing exchangeability, and atmospheric conditions suitable for conventional M(II)-Fe(III) LDHs may be inappropriate for green rust or redox-sensitive MnFe phases. Predictive synthesis must therefore be based on system-specific experimental domains rather than a generalized LDH rule.

Design of experiments appears to remain uncommon in the surveyed binary M-Fe LDH literature. Studies of related LDHs have shown that factorial and response-surface designs can quantify interactions among concentration, alkalinity, stirring, aging, hydrothermal treatment, and reactor configuration and relate them to particle size, aggregation, crystallinity, and surface area [108,231]. Similar approaches should be used first for variable screening and then for response-surface modeling of M-Fe systems. Relevant outputs should extend beyond performance to include measured solid composition, phase fractions, apparent coherent-domain dimensions, platelet morphology, wet-state aggregation, interlayer composition, and operationally defined accessible-site density. Models should report uncertainty and be tested using independently synthesized validation batches before being considered predictive beyond the original experimental domain.

Continuous and automated synthesis could support this transition by improving control over mixing, residence time, temperature, reagent delivery, and aging. Continuous-flow studies have already revealed process-dependent phase boundaries, including a temperature-controlled transition between NiFe-LDH and brucite-like products [113], while software-controlled platforms have demonstrated automated execution of LDH synthesis procedures [209,232]. Future systems should integrate pH-stat dosing, controlled precursor flow and micromixing, in-line pH, conductivity, or turbidity monitoring, and standardized aging and workup. Linking these process records to measured composition, phase fractions, particle dimensions, aggregation, and site accessibility would enable reproducible dataset generation and eventually closed-loop optimization.

Related hydroxide and oxide systems demonstrate that such mapping is feasible. Thermodynamically guided experiments have produced validated NH_3_–pH–temperature maps for multicomponent hydroxides, data-driven models have predicted iron oxide phase and particle size from synthesis conditions, and autonomous experimentation has identified nonequilibrium phase boundaries without exhaustive screening [233,234,235]. For M-Fe LDHs, thermodynamic calculations should be combined with controlled experiments because equilibrium models alone may not capture local supersaturation, kinetic trapping, precursor-mediated transformation, redox changes, or aging-dependent reorganization.

The quality of predictive maps will depend on the inclusion of unsuccessful as well as successful experiments. Published studies preferentially report optimized products, whereas amorphous precipitates, Fe-rich intermediates, off-target compositions, secondary phases, low yields, and irreproducible batches are rarely described in sufficient detail [236,237,238]. These outcomes define the boundaries of the accessible phase space and can improve prediction more than additional measurements near an already optimized condition. Negative results should therefore be retained with complete synthesis histories, measured compositions, phase assignments, product yields, and solution metal balances, and classified according to failure mode.

Uncertainty and reproducibility should be incorporated directly into the maps. Relevant uncertainties include pH control, reagent delivery, composition analysis, phase quantification, particle-size statistics, surface area, and accessible-site measurements. Repeated syntheses are needed to distinguish meaningful trends from batch variability, particularly when small differences in carbonate uptake, local pH, mixing, or oxidation state alter the final material. A synthesis condition should therefore be represented, where sufficient replicate data are available, by an outcome distribution and associated uncertainty rather than by a single idealized point.

Data-driven methods and machine learning may eventually complement thermodynamic modeling and design of experiments, but they cannot compensate for incomplete or inconsistent data. Related materials studies show that synthesis outcomes can be learned from structured experimental and literature-derived datasets [239]. For M-Fe LDHs, however, reports rarely provide machine-readable pH trajectories, addition histories, hydrodynamic descriptors, measured compositions, quantitative phase fractions, or unsuccessful outcomes [240]. Models trained predominantly on successful syntheses and nominal formulas would, therefore, risk learning reporting practices and publication bias rather than transferable phase-formation relationships. Metadata standardization is therefore a prerequisite, and interpretable regression or classification methods should be preferred for initially limited datasets before more complex algorithms are introduced.

Transferability must also be tested across laboratories. Differences in water quality, precursor purity, reactor geometry, mixing, CO_2_ exposure, workup, and storage can produce different materials from nominally identical procedures. Interlaboratory studies of related nanomaterials and porous solids have reported substantial variation in phase identity, particle dimensions, aggregation, defect content, and surface area [241,242]. Therefore, validation should distinguish batch-to-batch, operator-to-operator, reactor-to-reactor, and between-laboratory variability. A synthesis–structure model should be described as transferable only after successful external validation against predefined criteria appropriate to the predicted outputs, such as composition, phase fraction, yield, or nanostructural descriptors [243].

The immediate priority is therefore not artificial intelligence itself but the creation of standardized, information-rich, system-specific datasets. Progress requires coordinated use of multivariate experimental design, automated or continuous synthesis, time-resolved monitoring, quantitative structural descriptors, negative outcomes, uncertainty analysis, and external validation. These elements would allow the field to move from isolated optimized recipes toward predictive maps capable of targeting phase identity, composition, nanostructure, accessibility, and stability in binary Fe-containing layered hydroxides.

The main research gaps, priority evidence, and target deliverables are summarized in Table 6.

### 5.6. Conclusions

This review shows that the coprecipitation of binary Fe-containing layered hydroxides should not be treated as a single, universally reproducible synthesis route. Although the method is often described through nominal pH, M/Fe ratio, and aging time, the final material is also controlled by local supersaturation, reagent delivery, mixing efficiency, precursor speciation, complexation, atmosphere, carbonate availability, and post-precipitation treatment. Therefore, synthesis–structure relationships cannot be interpreted reliably from simplified recipes alone.

The commonly cited compositional interval of x = 0.20–0.33 is useful as an initial guideline for conventional M(II)-Fe(III) LDHs, but it should not be used as a universal stability window. Accessible compositional ranges and competing phases are system-specific and depend on metal identity, hydrolysis and redox chemistry, interlayer anion, precipitation conditions, and aging. Moreover, conventional M(II)-Fe(III) LDHs, CaFe hydrocalumite-/AFm-related layered hydroxides, and Fe(II)/Fe(III) green rust should be treated as structurally related but crystal-chemically distinct families rather than interpreted through a single compositional model.

The first solid phase formed during precipitation may not be the final LDH. Fe-rich intermediates, ferrihydrite-like precursors, sorption of the second metal, dissolution–reprecipitation, and oxidation-mediated transformation can all influence the final layered product. For this reason, final-product characterization cannot fully resolve formation pathways. Time-resolved analysis of both solid and solution phases is therefore required to distinguish direct mixed-metal formation from precursor-mediated and transformation-driven routes.

Nominal composition and an LDH-like PXRD pattern are likewise insufficient to establish homogeneous cation incorporation or phase purity. Feed composition may differ from the measured composition of the recovered solid, while diffraction broadening can reflect limited coherent-domain size, stacking disorder, microstrain, interstratification, or poorly crystalline and multiphase products. Phase assignment should therefore be claim-driven and supported by measured composition together with complementary evidence selected to resolve the relevant structural alternatives. In particular, agreement between nominal and bulk M/Fe ratios does not by itself demonstrate lattice-level incorporation of both metals into a common layered phase.

Nanostructure descriptors should not be used as direct substitutes for active-site accessibility. Platelet thickness, lateral dimensions, apparent coherent-domain size, BET surface area, pore architecture, aggregation, interlayer accessibility, local coordination, and defect-related structure represent different levels of material description and should not be used interchangeably. Thin nanosheets, high BET surface area, broad XRD reflections, mesoporosity, or flower-like aggregation may support a structure–performance discussion, but they do not independently demonstrate accessible or operationally active sites. Robust structure–reactivity relationships require progression from independently measured structural descriptors to chemically accessible environments and, where activity is claimed, to mechanism-relevant evidence of operational sites.

Fe-containing layered hydroxides should also be evaluated across relevant material states. Aging, washing, drying, storage, activation, reconstruction, oxidation, and regeneration may alter interlayer chemistry, aggregation, oxidation state, and phase identity. The as-synthesized, active/working, recovered, and regenerated materials should therefore be treated as potentially distinct structural states. Retention of performance or an LDH-like diffraction pattern after use does not by itself demonstrate preservation of the initial structure, just as regeneration of function does not necessarily imply restoration of the pristine material.

Taken together, these conclusions shift the central question from whether a nominal M-Fe LDH can be synthesized to whether its formation pathway, composition, phase identity, nanostructure, accessibility, and structural evolution can be demonstrated reproducibly. Progress toward predictive synthesis will require standardized, information-rich reporting, system-specific synthesis–composition–phase maps, time-resolved formation studies, quantitative structural and accessibility descriptors, reporting of negative as well as successful outcomes, uncertainty analysis, and independent validation. Such a framework can move the field from isolated optimized recipes and structure–performance correlations toward experimentally validated synthesis–structure relationships.

The synthesis and structural framework established here provide a basis for interpreting the environmental behavior of binary Fe-containing layered hydroxides. However, similar initial materials may follow different pathways depending on pollutant speciation, reaction conditions, water matrix, removal mechanism, transformation during use, recovery, and regeneration. These application-dependent mechanisms, cross-system environmental performance, real-water behavior, separation, reuse, and practical implementation fall outside the scope of the present synthesis-focused review and will be addressed in a companion Part II.

## Figures and Tables

**Figure 1:**
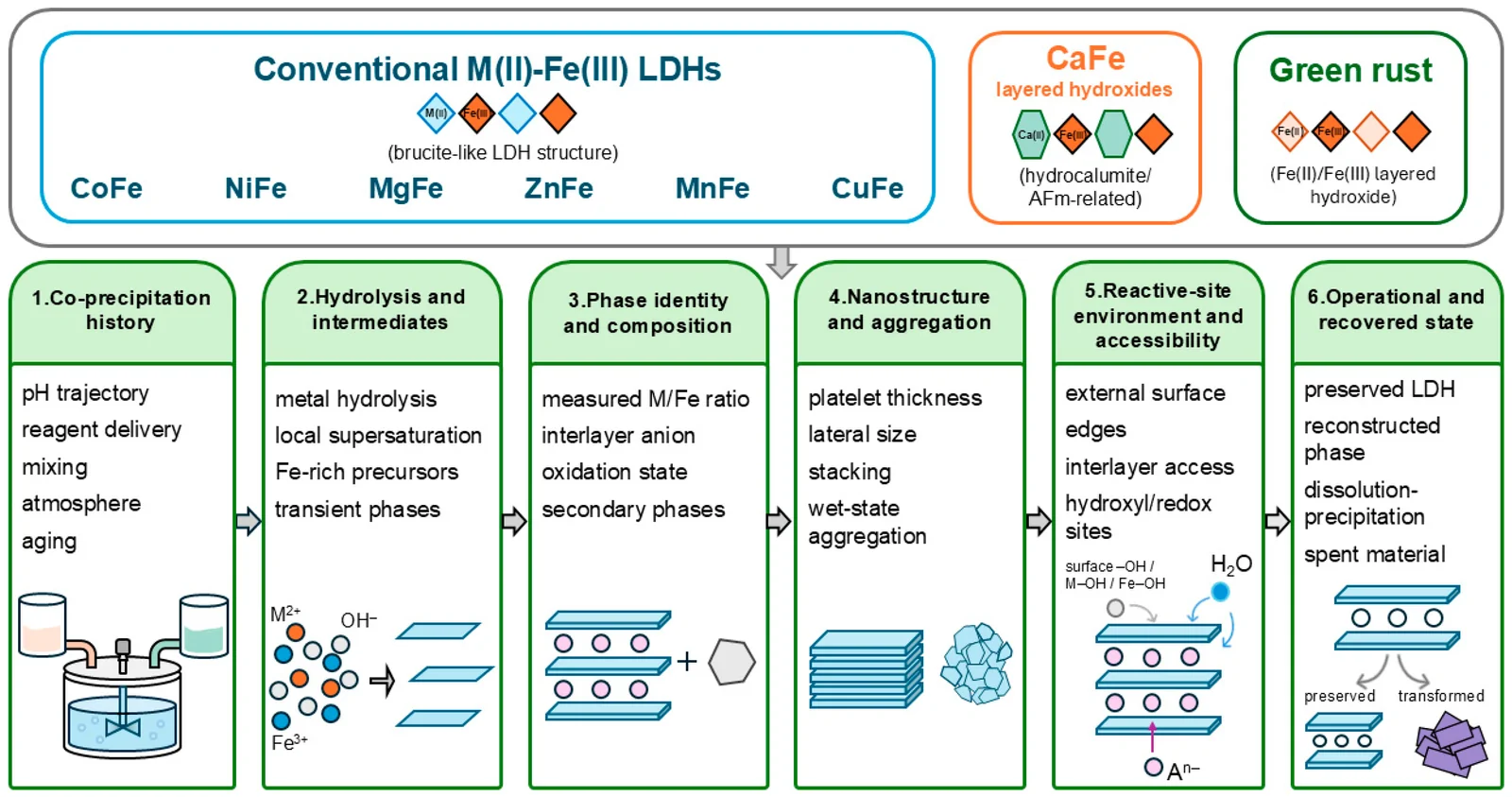
Conceptual framework of the review.

**Figure 2:**
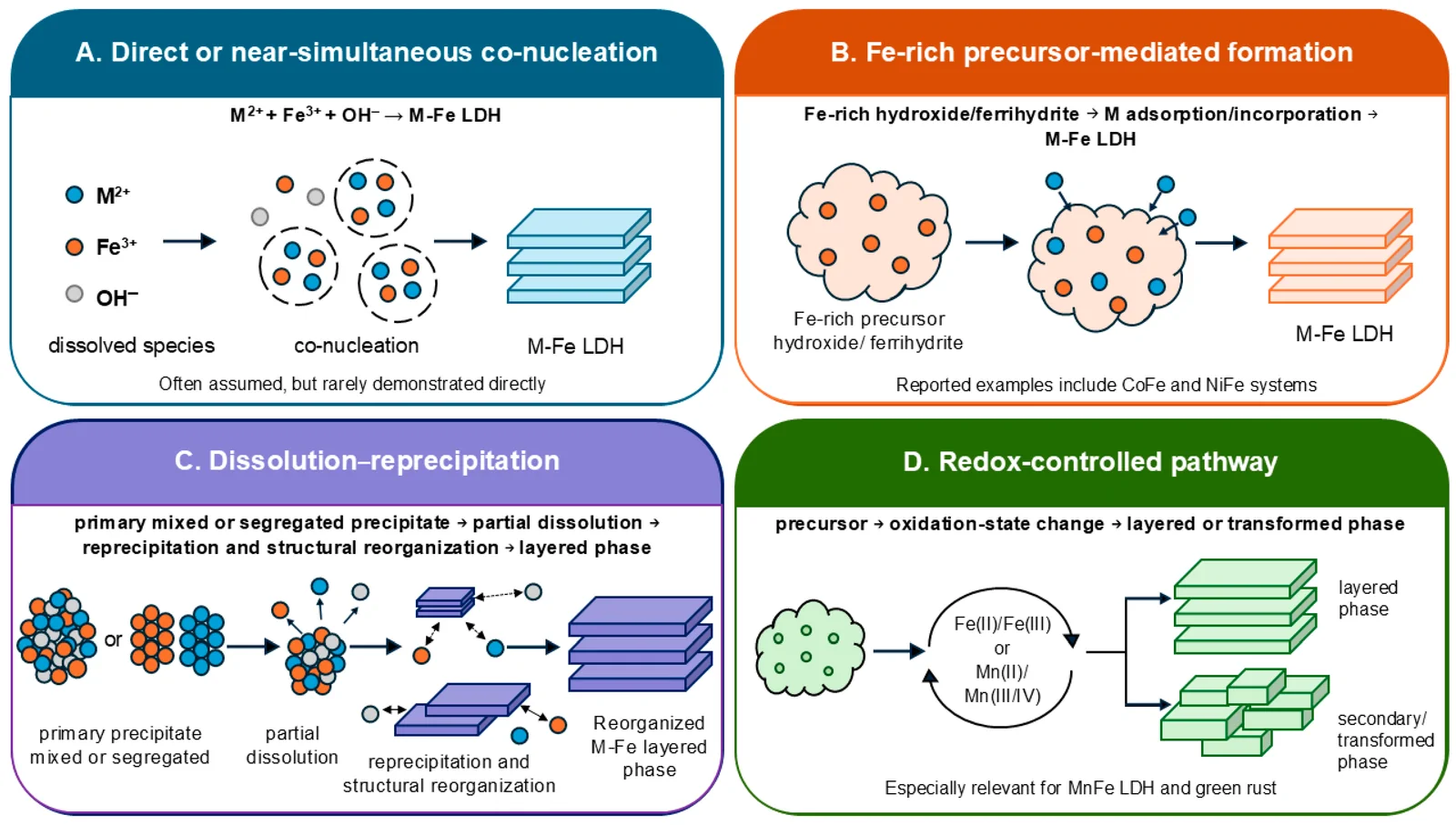
Alternative formation pathways of Fe-containing layered hydroxides.

**Figure 3:**
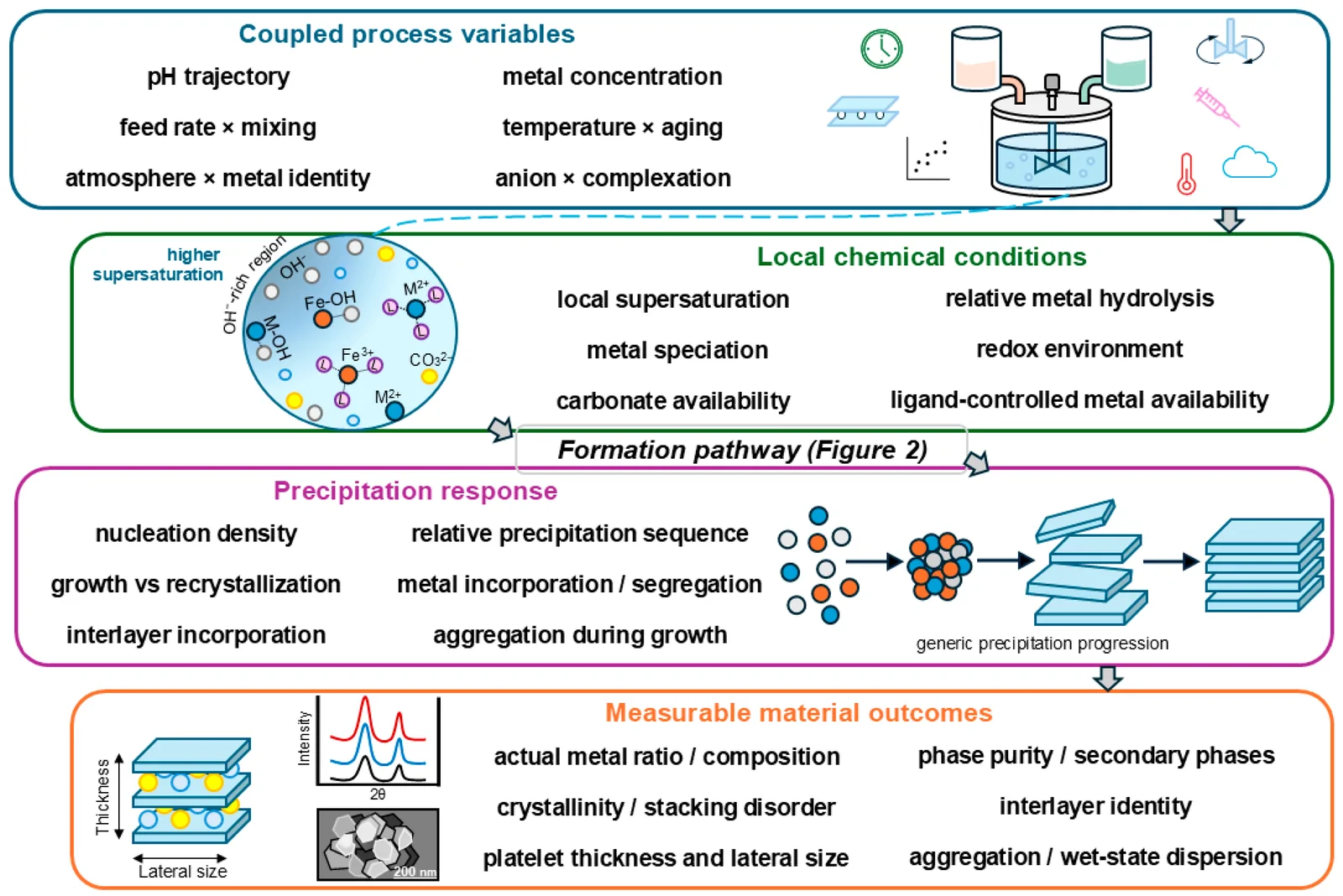
Coupled synthesis variables controlling phase formation and morphology.

**Figure 4:**
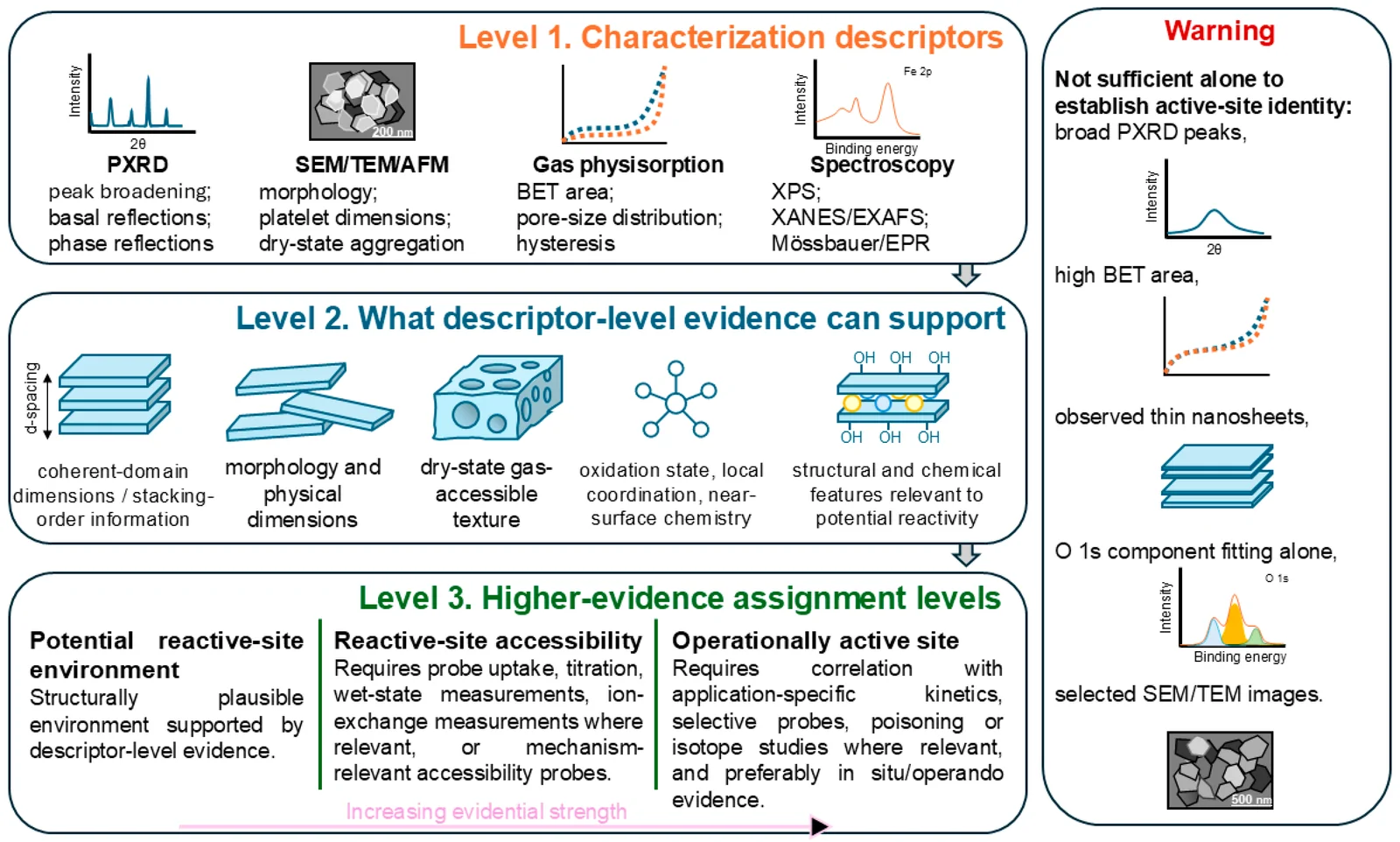
Evidence hierarchy for structural descriptors, reactive-site accessibility, and operational active-site assignment in Fe-containing layered hydroxides.

**Figure 5:**
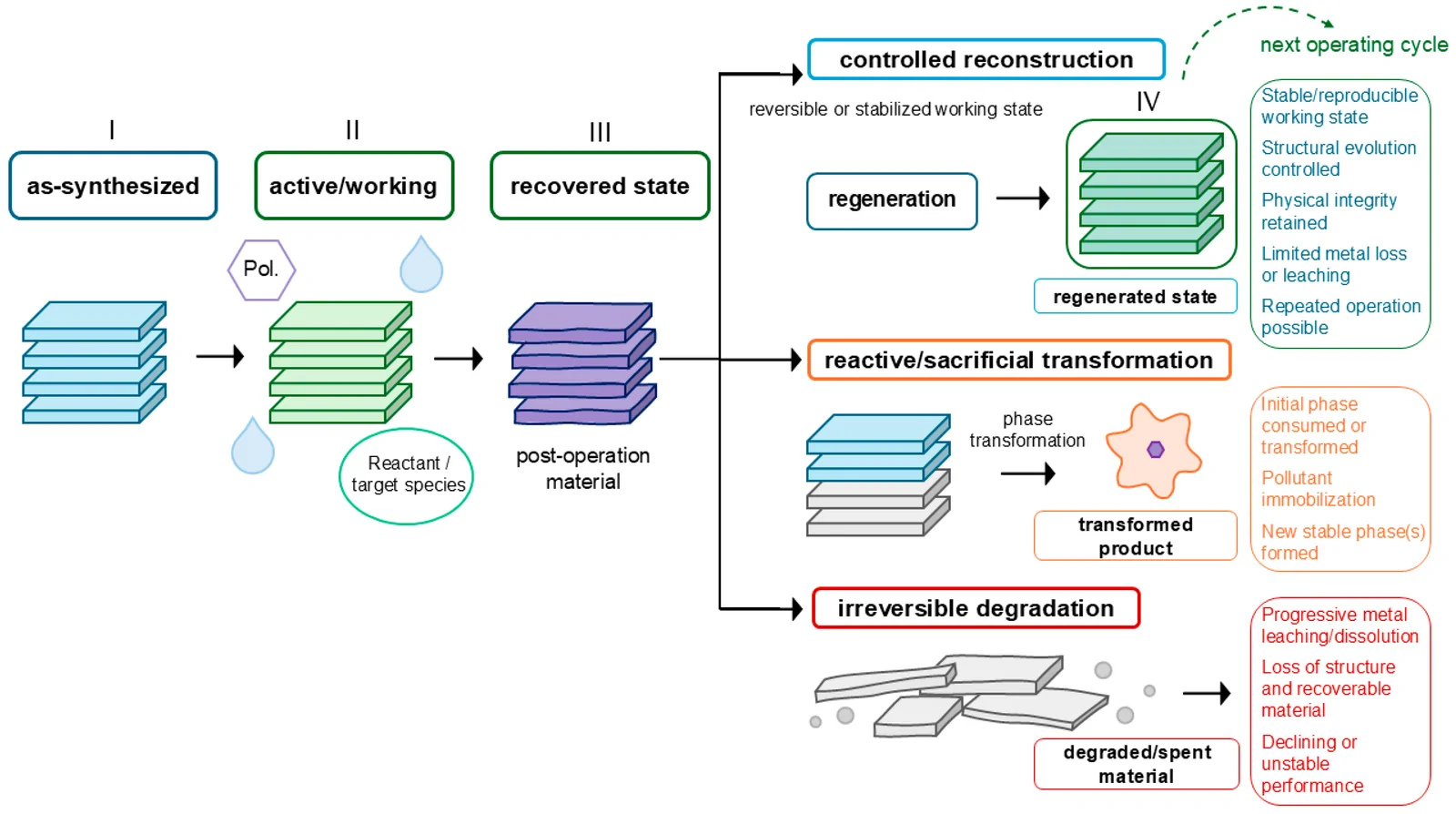
Conceptual framework distinguishing the as-synthesized, active, recovered or transformed, and regenerated states of Fe-containing layered hydroxides.

**Table 1:** System-specific crystal chemical features and phase-identification challenges of binary Fe-containing layered hydroxides.

System	Structural Family	Common Target Composition	Main Hydrolysis or Redox Complication	Typical Secondary-Phase Risk	Availability of Direct Formation Pathway Evidence	Priority Confirmation Methods
**CoFe**	Conventional LDH	x = 0.20–0.33; often Co/Fe = 2:1	Co oxidation, Fe-first hydrolysis	Ferrihydrite, Co oxyhydroxide	Relatively strong	XAS, Mössbauer, PDF
**NiFe**	Conventional LDH	x = 0.20–0.33	Fe-rich precursor, reconstruction	FeOOH, Ni(OH)_2_	Relatively strong	XAS, PDF, operando XRD
**MgFe**	Conventional LDH	x = 0.20–0.33	Sequential Fe hydrolysis	Fe-rich hydroxide/oxyhydroxide, brucite-like Mg(OH)_2_	Limited/moderate	Mössbauer, ICP balance
**ZnFe**	Conventional LDH	x = 0.20–0.33	ZnO or Zn hydroxysalts, depending on precursor and anion chemistry	ZnO, Zn hydroxysalts, Fe-rich phases	Limited	PDF, Raman, XAS
**MnFe**	Redox-sensitive LDH	No universal window established	Mn multivalence	Birnessite, MnFe_2_O_4_	Limited	Mn/Fe XAS, Eh monitoring
**CuFe**	Structurally uncertain conventional LDH	No universal window established	Jahn–Teller distortion, unequal hydrolysis	Cu(OH)_2_, CuO, Fe-rich amorphous phase	Very limited	Cu/Fe XAS, EPR, PDF
**CaFe**	Hydrocalumite-like	Ca/Fe ≈ 2 for idealized hydrocalumite/AFm-related layer stoichiometry	Precursor dissolution/speciation	Ca(OH)_2_, Fe hydroxide	Moderate	HR-XRD, PDF, solution balance
**Green rust**	Mixed-valent Fe layered hydroxide	Fe(II)/Fe(III)-dependent	Rapid oxidation	Magnetite, Fe(III) oxyhydroxides	Relatively strong	Mössbauer, XAS, anoxic in situ methods

Evidence strength refers to the availability of direct time-resolved, pH-resolved, in situ/operando, or otherwise phase-resolved studies of formation pathways, rather than to the overall number of publications on each material system.

**Table 2:** Comparative structural features of Fe-containing layered hydroxides.

Material Group	Structural Basis	Key Compositional or Redox Feature	Dominant Transformation Behavior	Main Interpretive Caution
Conventional M(II)-Fe(III) LDHs [38]	Brucite-like, edge-sharing M(OH)_6_ octahedral layers with partial substitution of M(II) by Fe(III)	System-dependent cation compatibility, ordering, redox sensitivity, and secondary-phase formation	System- and application-dependent ion exchange, surface reconstruction, dissolution, redox transformation, or conversion to oxyhydroxide-like phases	Not structurally interchangeable; the active phase may differ from that of pristine LDH
Ca-Fe layered hydroxides [40,42,64]	Hydrocalumite- or AFm-related layers with crystallographically distinct Ca and Fe environments	Narrower compositional flexibility and stronger deviation from a conventional brucite-like solid solution	Dissolution–precipitation, ion release, and formation of Ca-containing contaminant precipitates	Use “Ca-Fe layered hydroxide” unless an AFm- or hydrocalumite-like structure is confirmed
Fe(II)/Fe(III) green rust [2,67,80]	Mixed-valent Fe hydroxide layers containing structural Fe(II) and Fe(III)	Fe(II)/Fe(III) ratio controls reducing capacity and oxidation sensitivity	Oxidation of structural Fe(II) and transformation into Fe-rich oxides or oxyhydroxides	Handling and reaction may rapidly alter Fe speciation, phase identity, and reductive activity

**Table 3:** Effects of synthesis variables on precipitation chemistry, structure, and morphology of M-Fe layered hydroxides.

Synthesis Variable	Immediate Chemical Effect	Possible Structural Consequence	Main Confounding Factors	Representative Systems with Direct Evidence
pH trajectory	Metal hydrolysis and solubility	Phase identity, actual composition	Complexation, feed mode	CoFe, NiFe, ZnFe
Local supersaturation	Nucleation/growth balance	Coherent-domain size, aggregation, secondary phases	Mixing, addition rate	Mainly NiFe/CoFe
M/Fe ratio	Layer charge and cation population	Local ordering/segregation, secondary-phase risk	Actual solid composition	NiFe, CoFe, ZnFe
Mixing/feed position	Local OH^−^ and reagent exposure	Heterogeneous nucleation, Fe-rich intermediates	Reactor geometry	Limited direct M-Fe evidence
Temperature/aging	Transformation and recrystallization kinetics	Coherence, particle growth, phase transformation	pH, anion, redox state	NiFe, CaFe
Atmosphere	O_2_ and CO_2_ availability	Oxidation state, interlayer/phase identity	Handling and storage	MnFe, CaFe, green rust
Complexants	Metal speciation and delayed hydrolysis	Precursor pathway, composition, morphology	Ligand incorporation	NiFe, MnFe
Interlayer anion	Charge compensation and hydration	Basal spacing, stability, exchangeability	Carbonate contamination	NiFe, MgFe, CaFe

The listed systems represent those for which the reviewed literature provides comparatively direct evidence for the indicated synthesis-variable response. Their absence from a row does not imply that the corresponding variable is unimportant in other M-Fe systems.

**Table 4:** Evidence hierarchy for structural assignments and reactive-site accessibility in Fe-containing layered hydroxides.

Claimed Property	Evidence Often Used	Why It Is Insufficient When Used Alone	Recommended Complementary Evidence
**Small particle size**	Broad PXRD peaks	Peak broadening measures coherent-domain size and may also reflect strain, disorder, or stacking faults	TEM/AFM size statistics; diffraction analysis where appropriate
**Phase purity**	No additional PXRD peaks	Poorly crystalline or amorphous secondary phases may remain undetected	Bulk composition and complementary phase-sensitive methods such as Mössbauer, XAS, PDF, or spectroscopy
**Intercalation**	Shift of a single basal reflection	Basal spacing can also change because of hydration, stacking disorder, or reconstruction	XRD, vibrational spectroscopy, and interlayer anion/solution balance
**Ultrathin nanosheets**	Representative SEM image	SEM does not provide reliable nanosheet thickness and representative images do not establish a distribution	AFM step-height statistics and/or edge-on TEM
**Uniform slit-shaped pores**	H3 hysteresis	H3 hysteresis is non-unique and may arise from interparticle voids or platelet aggregation	Model-aware sorption analysis, microscopy and/or SAXS
**Oxygen vacancy or related defect assignment**	O 1s XPS fitting	Hydroxyls, adsorbed water, carbonate, deprotonated oxygen species, and different coordination environments can overlap	EPR/XAS, stoichiometric or structural evidence, and controlled defect comparison
**Potential reactive-site environments**	High BET area, thin nanosheets, defects, exposed edges, or fitted XPS components	Structural descriptors indicate plausible environments but do not establish that a specific chemical site exists or participates in reaction	Site-sensitive spectroscopy/local-structure characterization and structural confirmation
**Reactive-site accessibility**	BET surface area or nanosheet thickness	Dry N_2_-accessible area and morphology do not establish accessibility under hydrated operating conditions	Probe molecule uptake, titration, wet-state accessibility measurements, or mechanism-relevant adsorption/redox probes
**Operationally active sites**	Structural descriptors, defect assignment, or ex situ XPS alone	Site presence or accessibility does not demonstrate participation in a specific catalytic, adsorption, or electrochemical process	Application-specific kinetics/probes and in situ/operando evidence where feasible
**Stability**	Retained PXRD pattern after use	Surface reconstruction, local redox changes, dissolution, and metal leaching may occur without major changes in bulk diffraction	Dissolved metal balance and bulk and surface/phase characterization before and after operation

Potential reactive-site environments refer to structurally or chemically plausible sites, whereas reactive-site accessibility denotes their physical and chemical accessibility under relevant conditions. Assignment of an operationally active site requires application-specific evidence demonstrating its participation in the investigated process.

**Table 5:** Minimum and advanced reporting requirements for co-precipitated Fe-containing layered hydroxides.

Category	Minimum Reporting Information	Advanced Descriptors
Precursors	Chemical identity, purity, concentration	Speciation, relevant impurities
Dosing	Addition sequence, feed position, rate, duration	Time-resolved/automated dosing record
pH	Initial/final pH, set point where applicable, control mode, trajectory	Spatial or in-line monitoring
Reactor/mixing	Vessel geometry, working volume, impeller/stirrer type and dimensions, stirring rate	Mixing time, power input, hydrodynamic or CFD descriptors
Atmosphere	Air/inert/anoxic, CO_2_ control	Dissolved O_2_ and redox potential
Temperature/aging	Time and temperature	Sequential intermediate sampling
Workup	Washing, drying, storage	Wet-state preservation/characterization
Product	Yield, measured bulk composition, interlayer anion evidence, principal phase identity	Quantitative phase fractions, solution/material balance, local-structure analysis
Reproducibility	Number of independent synthesis batches and variability of key measured outputs	Operator- and laboratory-level validation

**Table 6:** Priority research gaps and deliverables for predictive synthesis of Fe-containing layered hydroxides.

Knowledge Gap	Current Limitation	Priority Evidence	Priority Systems	Target Deliverable
Reproducibility	Nominal recipe, final pH, and rpm reported without complete synthesis history	Complete synthesis history, independent batches, uncertainty of key outputs	All	Reproducible reporting and validation framework
Compositional windows	Nominal M/Fe ratios and LDH-like PXRD used as phase boundaries	Controlled composition series, measured composition, phase-sensitive analysis	CuFe, MnFe, CaFe, ZnFe	System-specific synthesis–composition–phase map
Formation pathways	Mechanisms inferred mainly from final dried products	Time-resolved solid and solution analysis	MgFe, ZnFe, MnFe, CuFe, CaFe	Validated formation pathway map
Wet-state nanostructure	Predominantly dried SEM/TEM and gas sorption descriptors	Quantitative dry- and wet-state morphology/aggregation measurements	All	Hydrated-state morphology and aggregation model
Defect assignment	O 1s fitting	Phase-specific local-structure and complementary defect-sensitive evidence	CoFe, NiFe, MnFe	Quantified defect–structure relationship
Reactive-site accessibility	BET area, morphology, and disorder used as accessibility proxies	Mechanism-relevant probes and independently constrained site populations	All	Operationally defined accessible-site density
Predictive synthesis	One-variable optimization and successful recipes	DoE, negative outcomes, uncertainty, replicate and external validation	All	Validated and transferable synthesis–structure map

## Data Availability

No new data were created or analyzed in this study. Data sharing is not applicable to this article.

## Abbreviations

The following abbreviations are used in this manuscript:

2D	two-dimensional
AFM	atomic force microscopy
AFm	alumina-ferric oxide-monosubstituted phase
BET	Brunauer–Emmett–Teller
BJH	Barrett–Joyner–Halenda
DFT	density functional theory
DLS	dynamic light scattering
EDS	energy-dispersive X-ray spectroscopy
EDTA	ethylenediaminetetraacetic acid
EPR	electron paramagnetic resonance
EXAFS	extended X-ray absorption fine structure
FTIR	Fourier-transform infrared spectroscopy
HR-XRD	high-resolution X-ray diffraction
HRTEM	high-resolution transmission electron microscopy
ICP	inductively coupled plasma
ICP-MS	inductively coupled plasma mass spectrometry
ICP-OES	inductively coupled plasma optical emission spectroscopy
IUPAC	International Union of Pure and Applied Chemistry
LDO	layered double oxide
NIR	near-infrared spectroscopy
NMR	nuclear magnetic resonance
OER	oxygen evolution reaction
PDF	pair distribution function
PXRD	powder X-ray diffraction
rpm	revolutions per minute
SAXS	small-angle X-ray scattering
SEM	scanning electron microscopy
SEM-EDS	scanning electron microscopy coupled with energy-dispersive X-ray spectroscopy
STEM-EDS	scanning transmission electron microscopy coupled with energy-dispersive X-ray spectroscopy
STEM-EELS	scanning transmission electron microscopy coupled with electron energy-loss spectroscopy
TEA	triethanolamine
TEM	transmission electron microscopy
XANES	X-ray absorption near-edge structure
XAS	X-ray absorption spectroscopy
XPS	X-ray photoelectron spectroscopy
XRD	X-ray diffraction
XRF	X-ray fluorescence
XRPD	X-ray powder diffraction
